# Human cytomegalovirus glycoprotein complex gH/gL/gO uses PDGFR-α as a key for entry

**DOI:** 10.1371/journal.ppat.1006281

**Published:** 2017-04-12

**Authors:** Yiquan Wu, Adrian Prager, Simone Boos, Moritz Resch, Ilija Brizic, Michael Mach, Sabrina Wildner, Laura Scrivano, Barbara Adler

**Affiliations:** 1 Max von Pettenkofer-Institute, Department of Virology, Ludwig-Maximilians-University Munich, Munich, Germany; 2 Institute for Clinical and Molecular Virology, Friedrich-Alexander University Erlangen-Nürnberg, Erlangen, Germany; Louisiana State University Health Sciences Center, UNITED STATES

## Abstract

Herpesvirus gH/gL envelope glycoprotein complexes are key players in virus entry as ligands for host cell receptors and by promoting fusion of viral envelopes with cellular membranes. Human cytomegalovirus (HCMV) has two alternative gH/gL complexes, gH/gL/gO and gH/gL/UL128,130,131A which both shape the HCMV tropism. By studying binding of HCMV particles to fibroblasts, we could for the first time show that virion gH/gL/gO binds to platelet-derived growth factor-α (PDGFR-α) on the surface of fibroblasts and that gH/gL/gO either directly or indirectly recruits gB to this complex. PDGFR-α functions as an entry receptor for HCMV expressing gH/gL/gO, but not for HCMV mutants lacking the gH/gL/gO complex. PDGFR-α-dependent entry is not dependent on activation of PDGFR-α. We could also show that the gH/gL/gO—PDGFR-α interaction starts the predominant entry pathway for infection of fibroblasts with free virus. Cell-associated virus spread is either driven by gH/gL/gO interacting with PDGFR-α or by the gH/gL/UL128,130,131A complex. PDGFR-α-positive cells may thus be preferred first target cells for infections with free virus which might have implications for the design of future HCMV vaccines or anti-HCMV drugs.

## Author summary

The identification of cellular receptors recognized by viral glycoproteins promoting entry is central for understanding virus pathogenesis and transmission for any virus. Although the roles of alternative gH/gL complexes of HCMV in cell tropism and virus spread have been extensively studied in cell culture, transfer to HCMV tropism *in vivo* is a controversial issue. Our characterization of the PDGFR-α –gH/gL/gO interaction offers an explanation for the tropism of HCMV for cells and tissues with high levels of surface PDGFR-α *in vivo*. Discrepant findings, when similar cell types were analyzed in culture, may retrospectively be attributed to a culture-dependent loss or up-regulation of PDGFR-α protein levels. Our finding that the PDGFR-α—gH/gL/gO interaction starts the predominant entry pathway for infection with free virus moves the gH/gL/gO complex in the center of interest for vaccines designed to prevent horizontal or vertical transmission and also for the development of CMV vaccine or gene therapy vectors.

## Introduction

Human cytomegalovirus (HCMV) is a human herpesvirus which is spread worldwide and can cause life-threatening infections in immunocompromised patients. Additionally, it is one of the major causes of virus-induced birth defects after congenital infection. Like all herpesviruses, it persists lifelong in its host. HCMV disease in immunocompromised patients and intrauterine infection with HCMV can be observed both after primary infection and after reactivation [1]. One hallmark of HCMV infection is the broad cell tropism observed *in vivo* [2], which is shaped by a number of different envelope glycoprotein complexes.

Initial attachment of HCMV particles to cells is promoted by heparan sulfate proteoglycans on the surface of cells [3]. Both, the HCMV glycoprotein gB and the HCMV gM/gN glycoprotein complex are involved in this initial attachment [4,5]. This step is believed to be followed by a more stable and specific interaction of cellular entry receptors with either the trimeric gH/gL/gO or the pentameric gH/gL/UL128,130,131A envelope glycoprotein complex [6]. Once the receptor—gH/gL complex interaction is stabilized, the core gH/gL complex in concert with gB is believed to promote fusion of the viral envelope with cellular membranes [6,7]. The *in vitro* phenotypes associated with a loss of the trimeric or the pentameric complexes are completely different. Mutants unable to form gH/gL/UL128,130,131A lose their broad cell tropism and classical host cells like endothelial, epithelial, monocytic or dendritic cells can no longer be infected *in vitro* [8–13]. Yet, the capacity to infect fibroblasts and virus production in fibroblasts is not affected [10,12]. Mutants unable to form gH/gL/gO or mutants with low amounts of gH/gL/gO in their envelopes primarily spread cell-associated, because gO-negative virus particles released from infected cells are hardly infectious [14–17]. Yet, their host cell range is not restricted [14,15]. Mutants unable to form either of the gH/gL complexes do not release infectious virus nor can they spread in a cell-associated manner [15]. The roles of the HCMV gH/gL complexes *in vivo* are not clear. A study on the role of the gH/gL/gO complex of murine cytomegalovirus (MCMV) showed that in primary infection, gH/gL/gO is crucial for infection of first target cells including epithelial cells, endothelial cells and macrophages [18]. Comparable to observations in cell culture [14,15,19,20], spread of infection from these first targets within organs is not dependent on gO as long as an alternative gH/gL complex can be formed [18].

Several studies indicated that gH/gL/gO and gH/gL/UL128,130,131A use distinct receptors for entry [20–22]. Until today, a number of different host cell surface molecules have been shown to enhance HCMV infection of cells in culture [23–28]. Additionally, it has been shown that binding of HCMV to some of them can result in activation of signaling pathways [23,27,29,30]. Among those signaling cell surface receptors are growth factor receptors like platelet-derived growth factor receptor-α (PDGFR-α), epidermal growth factor receptor (EGFR) or integrins [23,26–28]. PDGFR-α, EGFR, and integrins have been shown to bind gB or gH [23,27,28,31]. Recently, it has been shown that PDGFR-α binds recombinant gH/gL/gO [32].

Here, we could for the first time show that the gH/gL/gO complex in concert with gB binds PDGFR-α when HCMV virus particles attach to host cell surfaces. This confirms the interaction of recombinant gH/gL/gO with PDGFR-α reported recently [32]. We could also show that the PDGFR-α—gH/gL/gO interaction starts the predominant entry pathway for infection of fibroblasts with free virus. Cellular PDGFR-α expression levels determined whether infection was dependent on the gH/gL/gO or the alternative gH/gL/UL128,130,131A complex. Interestingly, infection of fibroblasts was not dependent on activation of PDGFR-α. By silencing PDGFR-α, we could show that the PDGFR-α –gH/gL/gO interaction not only promoted infection with free virus, but also cell-associated virus spread. The dominance of gH/gL/gO-driven entry in infections with supernatant virus suggests that the PDGFR-α—gH/gL/gO interaction may play a crucial role in horizontal infection with free virus from body fluids like urine or breast milk and thus be an interesting target for vaccines or antiviral drugs designed to prevent HCMV primary infection.

## Results

### Virion gH/gL/gO binds to PDGFR-α

**Fig 1:**
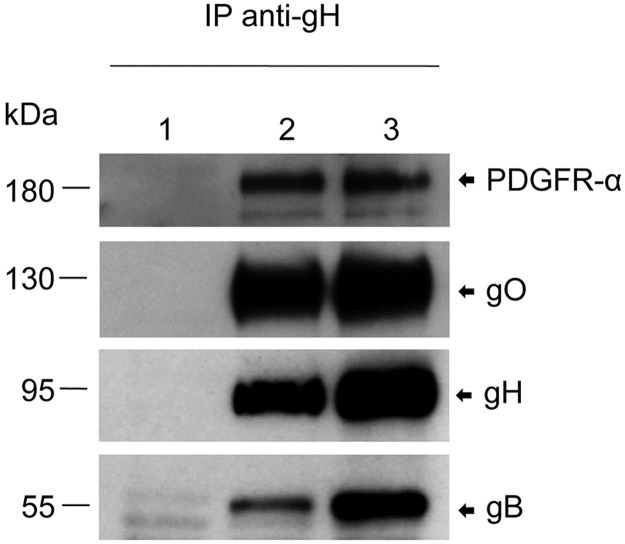
Anti-gH antibodies co-precipitate PDGFR-α, gO and gB from lysates of HFF co-incubated with TB40-UL131Astop virions. HFF surface proteins were biotinylated and lysates of HFF (1), lysates of HFF mixed with lysates of TB40-UL131Astop virions (2) and lysates of HFF co-incubated with TB40-UL131stop virions (3) were subjected to anti-gH immunoprecipitation. The precipitates were analyzed by Western blot using antibodies directed against PDGFR-α, gO, gH and gB.

**Fig 2:**
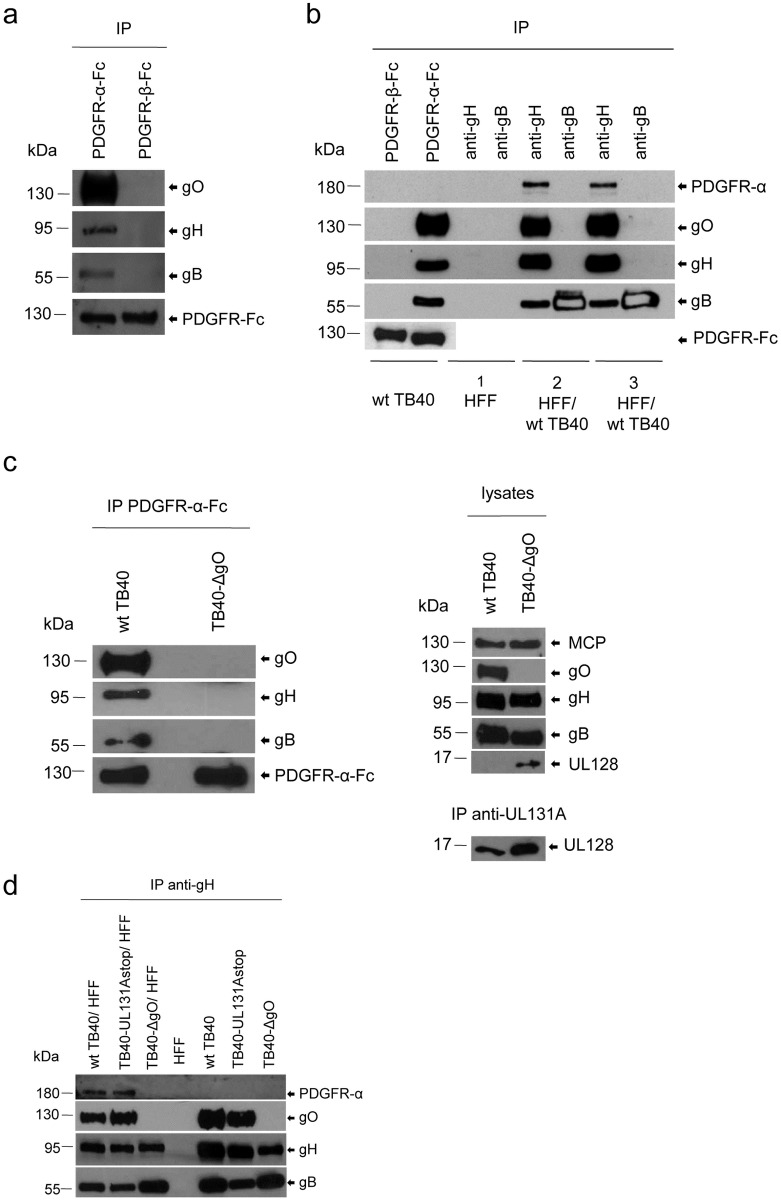
Co-precipitation of PDGFR-α and gH is dependent on gO. (a) Lysates of TB40-UL131Astop virions were co-incubated with PDGFR-Fc fusion proteins and then the Fc fusion proteins precipitated with protein A sepharose. (b) Lysates of wt TB40 virions were treated as described under (a). Additionally, lysates of HFF (1), lysates of HFF mixed with lysates of wt TB40 virions (2) and lysates of HFF co-incubated with wt TB40 virions (3) were subjected to anti-gH and anti-gB immunoprecipitation. Negatively stained gB bands in gB precipitations are due to abundant protein. (c) Left panel: Lysates of wt TB40 and TB40-ΔgO virions were co-incubated with PDGFR-α-Fc fusion protein and the Fc fusion proteins precipitated with protein A sepharose. Right panel: Lysates of wt TB40 and TB40-ΔgO virions adapted for equal amounts of MCP protein. UL128 was additionally precipitated from the lysates with a rabbit antiserum specific for HCMV UL131A. (d) Lysates of HFF mixed with lysates of virions, lysates of virions, or an HFF control lysate were subjected to anti-gH immunoprecipitation. The immunoprecipitates were analyzed by Western blot using antibodies directed against PDGFR-α, gO, gH and gB and the lysates under (c) additionally with antibodies directed against MCP and UL128.

It has been shown that recombinant gB [23] and recombinant gH/gL/gO [32] bind to cell surface PDGFR-α. This was interpreted as PDGFR-α being a cofactor for HCMV infection or PDGFR-α being an entry receptor, respectively. To find out whether PDGFR-α also interacts with these glycoprotein complexes in envelopes of HCMV particles, we co-incubated virus particles of the HCMV mutant TB40-UL131Astop [19], which is unable to form the gH/gL/UL128,130,131A complex, with surface-biotinylated human foreskin fibroblasts (HFF), lysed cells and surface-bound virions, and precipitated the HCMV glycoprotein gH with an anti-gH antibody. A streptavidin-stained blot of precipitated proteins showed a biotinylated cell surface protein of around 180 kDa which was co-precipitated from cell-virion lysates (S1 Fig, lane 3). This protein could also co-precipitated from mixtures of separately lysed HFF and virions (S1 Fig, lane 2), but not from HFF control lysates (S1 Fig, lane 1). The corresponding gel slices from a silver-stained gel (S1 Fig) were analyzed by mass spectrometry which showed one prominent hit for the cell-virion lysate, peptide KLVYTLTVPEATVKD, which matches the sequence of human PDGFR-α. Co-precipitation of PDGFR-α and gH could be confirmed by Western blot analysis (Fig 1). As expected, anti-gH antibodies co-precipitated the gH/gL/gO component gO [33,34] and also, as reported before, gB [7,35]. Using recombinant PDGFR-α-Fc fusion protein, we could co-precipitate gH, gO and gB from lysates of TB40-UL131Astop virions, whereas a control PDGFR-β-Fc protein did not bind any of these HCMV glycoproteins (Fig 2a).

Co-precipitation of gH, gO and PDGFR-α and additionally gB could either reflect an interaction of the gH/gL/gO complex with gB bound to PDGFR-α or interactions of gH/gL/gO with PDGFR-α and independently also with gB. To clarify the role of gO in interactions of gB and gH with PDGFR-α, we compared precipitations of gH, gO, gB and PDGFR-α from lysates of wildtype (wt) TB40 virus, which contains both, the trimeric and the pentameric gH/gL complex, with precipitations from lysates of a TB40 mutant lacking gO (TB40-ΔgO) [19]. As observed for TB40-UL131Astop virus, anti-gH antibodies co-precipitated gO and gB from cell-free wt TB40 virion lysates and additionally PDGFR-α from lysates of wt TB40 virions mixed with HFF (Fig 2b). PDGFR-α-Fc also co-precipitated gO, gH and gB from cell-free wt TB40 virion lysates (Fig 2b). Precipitation of gB with an anti-gB antibody did not result in co-precipitation of gH or gO or PDGFR-α (Fig 2b). Comparable failures of different anti-gB antibodies to precipitate complexes formed between recombinant gH/gL and gB have been described before and have been attributed to masking of gB epitopes by bound gH/gL complexes [7,35]. For a direct comparison of wt TB40 and TB40-ΔgO virions, the virion lysates used for co-precipitation assays were adjusted to contain equal amounts of HCMV major capsid protein (MCP) as a measure for equal numbers of particles (Fig 2c, right panel). TB40-ΔgO virions contained more UL128 protein than wt TB40 virions. In wt TB40 virions, UL128 could only be detected after enrichment by anti-UL131A co-precipitation (Fig 2c, right panel). As described before for the HCMV strain AD169 [12], gH proteins in lysates of cells infected with wt TB40 or TB40-ΔgO virus ran as double bands in the Western blot, whereas in lysates of purified virions only the respective upper bands could be detected (S2 Fig). gH from TB40-ΔgO virions, which contain only gH/gL/UL128,130,131 complexes, had a slower electrophoretic mobility than gH from wt TB40 virions, which predominantly contain gH/gL/gO complexes [36] (Fig 2d and S2 Fig). A similar size difference for gH has been described for extracts of cells expressing either recombinant gH/gL/gO or recombinant gH/gL/UL128,130,131A and thus resembling extracts of wt TB40 or TB40-ΔgO virions, respectively [21]. Although wt TB40 and TB40-ΔgO lysates contained comparable amounts of gH and gB, PDGFR-α-Fc could only co-precipitate gH, gO and gB from lysates of wt TB40 virions, but not from lysates of TB40-ΔgO virions (Fig 2c, left panel). This indicates that gH and gB can only interact with PDGFR-α when a gH/gL/gO complex is formed. In the same line, anti-gH antibodies failed to co-precipitate PDGFR-α from lysates of TB40-ΔgO virions mixed with lysates of HFF (Fig 2d). Fig 2d additionally shows that independently of whether cell-free wt TB40, TB40-UL131Astop, or TB40-ΔgO virions were analyzed or whether virions mixed with cells were analyzed, anti-gH antibodies co-precipitated gB. This probably reflects the recently described interaction of gH/gL complexes with gB in virions [35]. In summary, our co-precipitation data imply that the observed PDGFR-α –gH interaction is strictly dependent on gO which is indicative of a direct interaction of gH/gL/gO with PDGFR-α. An interaction of gB and PDGFR-α can only be observed when gH/gL/gO is formed which suggests that gB either binds to PDGFR-α after gH/gL/gO has formed a complex with PDGFR-α or that a preformed gB—gH/gL/gO complex binds to PDGFR-α. A recent publication by Vanarsdall et al., which showed that gB does not interact with gH/gL/gO in virions [35], rather supports the first scenario. Our data confirm the interaction of recombinant gH/gL/gO with PDGFR-α [32], but not a gH/gL/gO-independent interaction of gB with PDGFR-α [23].

Different host cells of HCMV show different levels of PDGFR-α expression [37]. We could easily detect PDGFR-α in extracts of fibroblasts (HFF and MRC-5), but not in extracts of 293 cells, ARPE-19 cells, or HUVEC (S3a Fig). Using a recombinant HCMV gO—mouse IgG2b-Fc fusion protein (gO-Fc) expressed in 293 cells and purified from cell culture supernatants (S3b Fig), we tested binding of gO to the cell surface of HCMV host cells. When gO-Fc was co-incubated with HFF or HUVEC, a strong and specific binding to HFF but not to HUVEC was observed (S3c Fig) which correlates with the respective PDGFR-α expression levels in these cells. This supports the recently described direct interaction of gO with PDGFR-α [32].

### Infection of fibroblasts with an HCMV gO-knockout mutant is not dependent on PDGFR-α

The co-precipitation data indicate that PDGFR-α only interacts with gH when virions contain gO. To study whether PDGFR-α and gO are indeed co-players in the same entry pathway, we compared infections with wt TB40 and TB40-ΔgO viruses in several independent experiments. Infections with wt TB40 and TB40-ΔgO viruses are both dependent on gB and gH which could be deduced from neutralization assays using anti-gB and anti-gH antibodies (S4a Fig). Since TB40-ΔgO virus is highly attenuated when compared to wt TB40 virus [15], infections with wt TB40 and TB40-ΔgO viruses had to be adjusted such that they resulted in equal numbers of IE1-positive cells after 24 hours (S4b and S4c Fig). We observed an approximately 1000-fold reduced infectivity for fibroblasts for TB40-ΔgO virus when infection efficiencies and MCP contents of virus preparations of wt TB40 and TB40-ΔgO viruses were compared.

**Fig 3:**
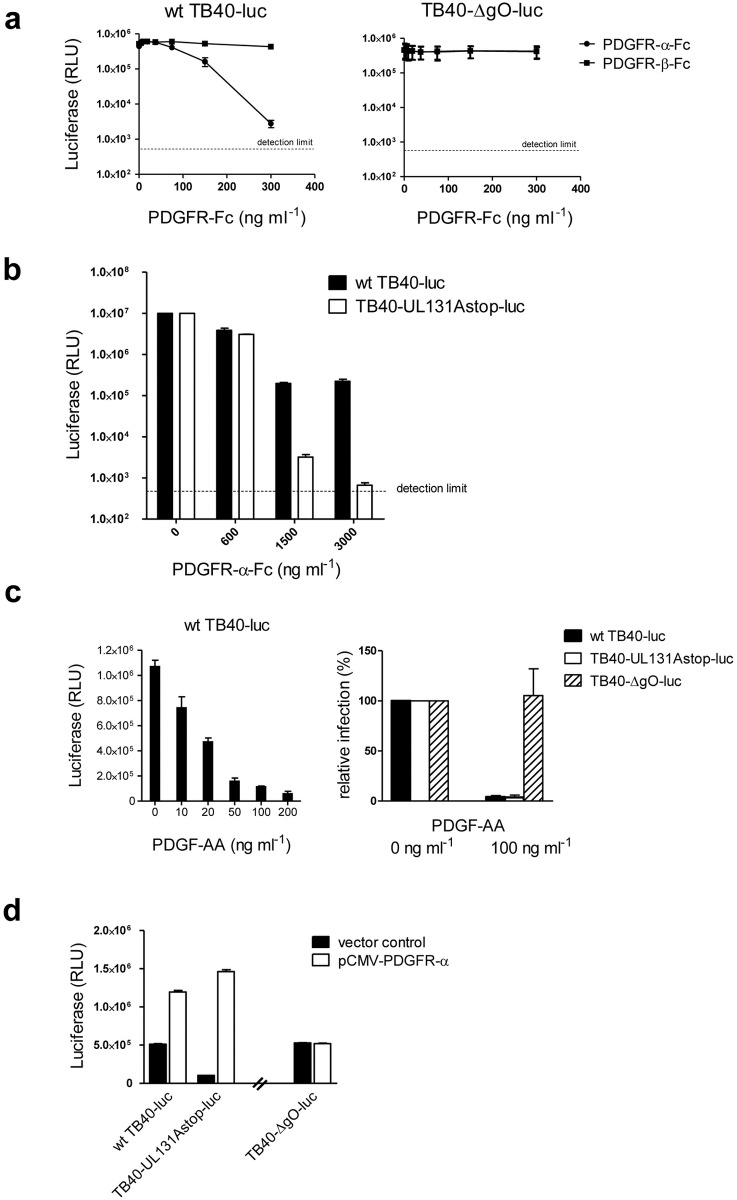
PDGFR-α-dependent and gO-dependent entry are congruent. HFF (a) to (c) or 293 cells (d) were infected on 96 well plates. (a) Before infection, wt TB40-luc or TB40-ΔgO-luc virus were pre-incubated with the indicated amounts of PDGFR-α-Fc or as a control PDGFR-β-Fc for one hour at 4°C. Cells were infected with the virion-PDGFR-Fc mixtures using centrifugal enhancement, washed three times with PBS and then incubated for 24 hours before infection was determined by a luciferase assay. (b) The experiment was performed as described under (a) with the difference that infections with wt TB40-luc and TB40-UL131stop-luc virus were compared and that cells were infected with a 50fold higher m.o.i. (c) Before infection, cells were pre-incubated with the indicated amounts of PDGF-AA for one hour at 4°C. Cells were then infected in the presence of PDGF-AA. After infection, free virus and cell surface-bound virus were inactivated by washing the cells three times with PBS, pH 3.0. 24 hours after infection cells were analyzed by a luciferase assay. The left panel shows inhibition of wt TB40-luc infection in the presence of increasing concentrations of PDGF-AA and the right panel shows inhibition of wt TB40-luc, TB40-UL131stop-luc, and TB40-ΔgO-luc viruses after infection of HFF pre-incubated with 0 ng ml^-1^ PDGF-AA (set to 100%) and with 100 ng ml^-1^ PDGF-AA. (d) 293 cells were transfected with pCMV-PDGFR-α or a control vector and 2 days later infected with wt TB40-luc, TB40-UL131Astop-luc or TB40-ΔgO-luc viruses. 24 hours after infection, cells were analyzed by a luciferase assay. (a), (c), and (d) show means +/- SD of three independent experiments done in triplicates. (b) and the left panel of (c) show one representative experiment done in triplicates.

In a first set of experiments, we pre-incubated virus preparations with soluble PDGFR-α-Fc fusion protein and as a control with PDGFR-β-Fc. PDGFR-α-Fc specifically and nearly completely inhibited infection of HFF with wt TB40 virus but not infection with TB40-ΔgO virus (Fig 3a). Interestingly, a residual infection of about 0.5% was observed when wt TB40 virus was co-incubated with PDGFR-α-Fc which matches the 1000-fold difference in infectivity between wt and gO-negative TB40 virions. Thus, blocking PDGFR-α –gH/gL/gO interactions results in a phenotype comparable to deletion of gO. The residual infection observed after pre-incubation of wt TB40 virus with PDGFR-α-Fc very likely reflects infection through a gH/gL/UL128,130,131A-dependent entry pathway. To show this, we compared inhibition of wt TB40 and TB40-UL131Astop virus infections by PDGFR-α-Fc. We started at a higher multiplicity of infection (m.o.i). to facilitate discrimination of the inhibition levels. This way, we could show that infection with TB40-UL131Astop virus, which can only form the gH/gL/gO complex, could be completely inhibited, whereas even increasing amounts of PDGFR-α-Fc could not abolish the residual infection of wtTB40 virus (Fig 3b). To exclude, that abundant TB40-ΔgO virus particles prevented inhibition of TB40-ΔgO virus just by unspecifically binding PDGFR-α-Fc protein, TB40-UL131Astop virus was mixed with TB40-ΔgO virus and inhibition by PDGFR-α-Fc analyzed. As before, infection with TB40-UL131Astop virus could be completely inhibited and infection with TB40-ΔgO virus could not be inhibited. When both virus preparations were mixed, infection was reduced to the level of the TB40-ΔgO infection, indicating that also in the presence of abundant TB40-ΔgO virus particles, infection with gH/gL/gO-positive wt TB40 virions could specifically be inhibited (S5 Fig).

It has been shown before that co-incubation of fibroblasts with the PDGFR-α ligand PDGF-AA blocks infection of fibroblasts with HCMV [23]. We co-incubated HFF with PDGF-AA and then infected the cells with wt TB40, TB40-UL131Astop, or TB40-ΔgO virus. PDGF-AA, which dose-dependently blocked infection of fibroblasts with wt TB40 virus (Fig 3c (left panel)), only inhibited infections with wt TB40 and TB40-UL131Astop viruses, but not infection with TB40-ΔgO virus (Fig 3c (right panel)).

In a recent publication, it has been shown that infection of ARPE-19 epithelial cells with the HCMV strain TR, which has both gH/gL complexes, can be strongly enhanced by over-expression of PDGFR-α in ARPE-19 cells [37]. We over-expressed PDGFR-α in 293 cells (S6a and S6b Fig) and studied the effect on infections with wt TB40, TB40-UL131Astop, and TB40-ΔgO viruses. wt TB40 or TB40-UL131Astop viruses were used at an equal m.o.i. (titrated on HFF) and TB40-ΔgO was adjusted such that an infection comparable to the wt TB40 infection of empty vector-transfected 293 cells was achieved (Fig 3d). Over-expression of PDGFR-α strongly enhanced infections with wt TB40 and TB40-UL131Astop viruses (Fig 3d and S6c and S6d Fig) but not infection with TB40-ΔgO virus (Fig 3d). This indicates that an increased PDGFR-α level can only enhance infections with gO-positive HCMV. As expected, TB40-UL131Astop virus, which lacks the pentameric complex, could less efficiently infect non-transfected 293 cells than wt TB40 virus (Fig 3d). Taken together, all three sets of experiments (Fig 3) clearly show that PDGFR-α-dependent and gO-dependent entry are congruent.

### PDGFR-α-dependent infection of fibroblasts does not depend on activation of PDGFR-α

**Fig 4:**
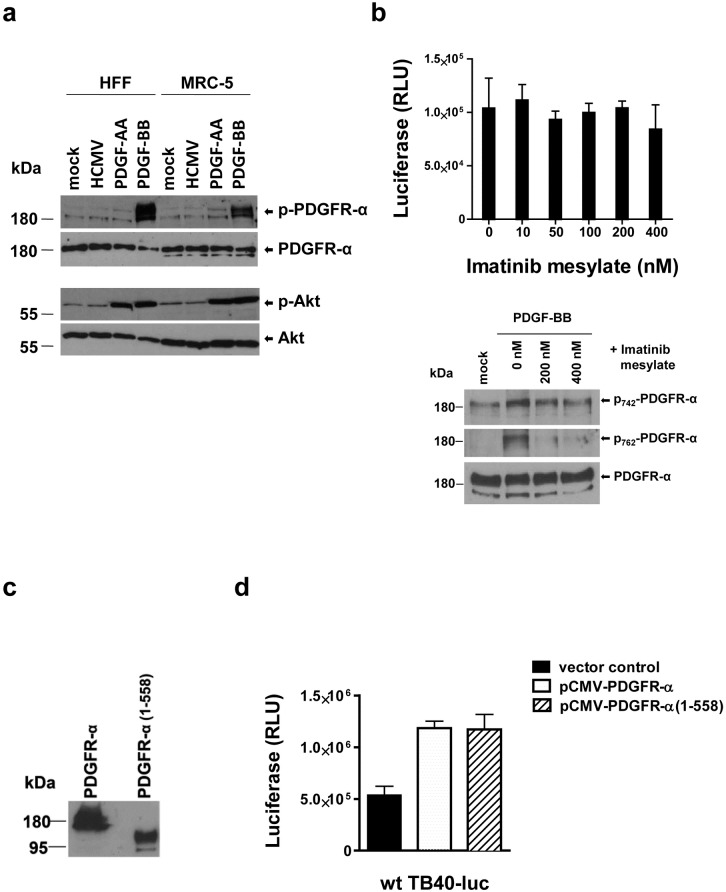
HCMV infection of fibroblasts is not dependent on phosphorylation of PDGFR-α. (a) Serum-starved HFF or MRC-5 were co-incubated with wt TB40 (m.o.i of 10 in 0.05% DMEM), PDGF-AA (100 ng ml^-1^), PDGF-BB (100 ng ml^-1^) or 0.05% DMEM (mock) for 1 hour at 4°C and then shifted to 37°C for 20 min. p-PDGFR-α or PDGFR-α and p-Akt or Akt were detected by Western blot analysis of total cell lysates. (b) HFF were pre-incubated with different concentrations of imatinib mesylate for 1 hour at 37°C and then infected with wt TB40-luc (m.o.i. of 1) for 90 min. Free virus and cell surface-bound virus were inactivated by washing the cells three times with PBS, pH 3.0. After 24 hours in medium containing the respective amounts of imatinib mesylate, a luciferase assay was performed to determine infection of cells. One representative experiment done in triplicates is shown. To control for the activity of imatinib mesylate, HFF were in parallel pre-treated with the indicated concentrations of imatinib mesylate for 1 hour at 37°C and then stimulated with PDGF-BB (100 ng ml^-1^) or mock-treated for 20 min. p_742_- and p_762_-PDGFR-α or PDGFR-α were detected by Western blot analysis of total cell lysates. (c,d) 293 cells were transfected with pCMV-PDGFR-α, pCMV-PDGFR-α(1–558), or a control vector. (c) Total cell extracts of pCMV-PDGFR-α- or pCMV-PDGFR-α(1–558)-transfected cells were analyzed for PDGFR-α expression by Western blot 24 hours after transfection using an antibody recognizing the N-terminus of human PDGFR-α (2D2-1A11). (d) 48 hours after transfection 293 cells were infected with wt TB40-luc. 24 hours after infection, cells were analyzed by a luciferase assay. Shown are means +/- SD of three independent experiments done in triplicates.

It has been reported that co-incubation of human embryonic lung fibroblasts (HELF) with HCMV virions results in phosphorylation of PDGFR-α and downstream Akt [23]. Similarly, co-incubations of MRC-9 cells with AD169 and VR1814 virions have been shown to result in a weak tyrosine phosphorylation of PDGFR-α [32]. Both studies concluded that PDGFR-α-dependent entry is indispensably linked to activation of PDGFR-α. We studied phosphorylation of PDGFR-α and downstream Akt in HFF and in a lung fibroblast cell line (MRC-5). Cells were co-incubated with HCMV and as controls with the PDGFR-α ligands PDGF-AA and PDGF-BB. To exclude an FCS-driven phosphorylation of PDGFR-α, we used a virus preparation which contained only very low levels of FCS. Both, PDGF-AA and PDGF-BB induced phosphorylation of Akt, but only PDGF-BB induced detectable phosphorylation of PDGFR-α (Fig 4a). Yet, we could not observe phosphorylation of PDGFR-α or Akt when HFF or MRC-5 fibroblasts were co-incubated with HCMV at an m.o.i. of 10 (Fig 4a). The failure of PDGF-AA to efficiently induce phosphorylation of PDGFR-α in dermal fibroblasts has been described before [38] and turned out here also to be a property of MRC-5 cells. Independently of these cell type-specific phosphorylation patterns, our data show that, although HCMV binds to PDGFR-α on the surface of fibroblasts, it does not necessarily activate it. In line with this, we did also not observe an inhibition of infection when HFF were pretreated with the protein kinase inhibitor imatinib mesylate, although we could show that it interfered with phosphorylation of PDGFR-α (Fig 4b). If PDGFR-α-driven entry was independent of activation of PDGFR-α, signaling-incompetent PDGFR-α and intact PDGFR-α should equally be able to enhance the susceptibility of host cells to HCMV infection. Using site-directed mutagenesis, we introduced a stop codon at amino acid position 559 in pCMV-PDGFR-α which resulted in a truncated protein consisting of the extracellular domain and the transmembrane anchor of PDGFR-α but lacking the cytoplasmic kinase domains (S7a Fig and Fig 4c). After transfection of 293 cells, both proteins were equally expressed on the surface of transfected 293 cells (S7b Fig). Over-expression of full-length PDGFR-α and truncated PDGFR-α equally enhanced wt TB40 virus infections of 293 cells which strongly suggests that PDGFR-α rather acts as a fusion-triggering receptor than as a signaling ligand (Fig 4d).

### gH/gL/gO-dependent cell-associated modes of HCMV spread are also PDGFR-α-dependent

**Fig 5:**
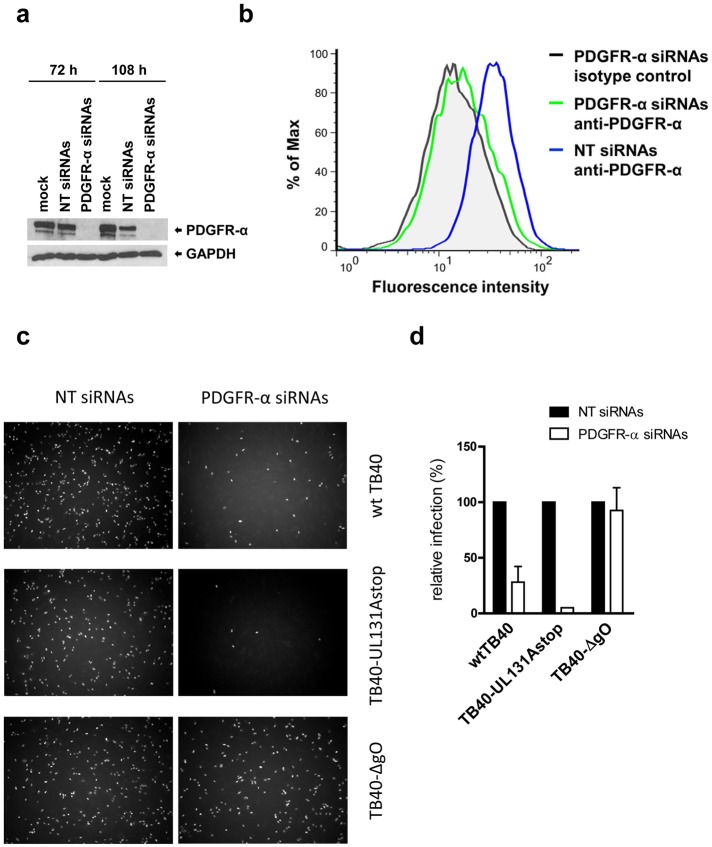
Silencing of PDGFR-α reduces infection of fibroblasts with gH/gL/gO-positive HCMV. HFF were transfected with PDGFR-α siRNAs, non-targeting (NT) siRNAs or mock-transfected. (a) 72 and 108 hours after transfection, total cell lysates were analyzed by Western blot for the expression of PDGFR-α or GAPDH. (b) 72 hours after transfection, cell surfaces of siRNA-transfected cells were stained with an anti-PDGFR-α antibody (35248) and a secondary Fluor 488-labelled anti-mouse antibody and analyzed by FACS. (c) 48 hours after transfection, cells were infected with wt TB40, TB40-UL131Astop and TB40-ΔgO viruses and 24 hours post infection stained for HCMV IE1 by indirect immunofluorescence. (d) HFF were infected as described under (c) and the percentage of IE1-positive nuclei was determined. Infection of cells transfected with NT siRNAs (set to 100%) and infection of PDGFR-α-silenced HFF is shown. Shown are means +/- SD from two independent experiments done in triplicates.

For HCMV and other herpesviruses, there is an ongoing discussion whether cell-free and cell-associated modes of virus spread are dependent on interactions of the same virion glycoproteins and cellular receptors or whether different protein-protein interactions or direct fusions between cells drive cell-associated virus spread [39–41]. In cell culture, cell-associated virus spread is defined as spread in the presence of neutralizing antibodies. However, it is not clear whether antibody-resistant spread reflects spread promoted by cell—virion interactions not recognized by the neutralizing antibodies or whether it reflects a failure of these antibodies to access viral envelope glycoproteins or neutralize locally concentrated virions when virions are transmitted from infected cells to neighboring cells. One approach to clarify this would be to knockdown the cellular receptor instead of blocking the receptor binding proteins. Pooled PDGFR-α siRNAs efficiently silenced PDGFR-α expression in HFF (Fig 5a and 5b). Silencing of PDGFR-α strongly reduced infections of HFF with cell-free wt TB40 or TB40-UL131Astop viruses, but not infection with TB40-ΔgO virus (Fig 5c and 5d) which confirmed our inhibition experiments using recombinant PDGFR-α or PDGFR-α ligand to block infection.

**Fig 6:**
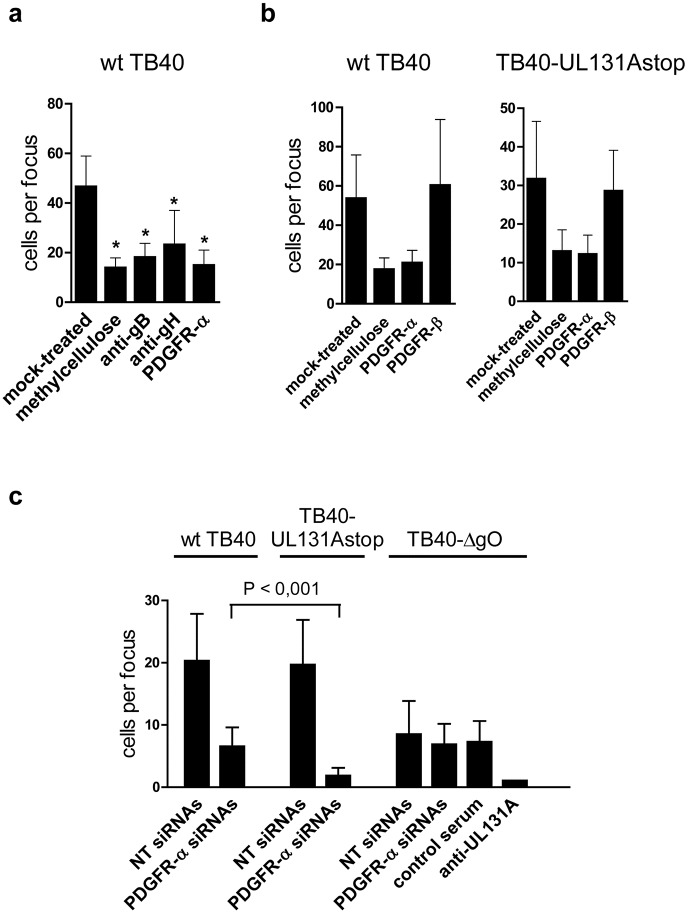
Silencing of PDGFR-α reduces cell-associated spread of gH/gL/gO-positive HCMV. (a) Confluent monolayers of HFF were infected with wt TB40 virus at a very low m.o.i. After infection, cells were either overlaid with methylcellulose or medium containing anti-gB antibodies (SM5-1, 2μg ml^-1^), anti-gH antibodies (14-4B), PDGFR-α-Fc (2 μg ml^-1^), or no inhibitor (mock-treated). (b) HFF infected with wt TB40 or TB40-UL131Astop virus were mixed with uninfected cells. After adherence, cells were either overlaid with methylcellulose or fresh medium was added containing 2 μg ml^-1^ PDGFR-α-Fc, 2 μg ml^-1^ PDGFR-β-Fc, or no inhibitor (mock-treated). (a,b) 5 days later, cells were stained for HCMV IE1 by indirect immunofluorescence and cells per focus counted. For each treatment, at least 10 (a) or 20 (b) foci were counted and depicted as means +/- SD. Shown are representative experiments. Asterisks under (a) represent P<0.001 values determined by comparing foci in mock-treated monolayers with foci in monolayers overlaid with methylcellulose, co-incubated with antibodies, or co-incubated with PDGFR-α-Fc (Mann-Whitney Rank Sum test). (c) NT siRNA or PDGFR-α siRNA-transfected HFF 48 hours after transfection were mixed with HFF infected with wt TB40, TB40-UL131Astop, or TB40-ΔgO virus. After adherence, cells were overlaid with methylcellulose or methylcellulose containing anti-UL131A rabbit antiserum (1:40) or a control rabbit antiserum (1:40). 5 days later, cells were analyzed as described under (a). The P value shown (Mann-Whitney Rank Sum test) was determined by comparing cell spread of wt TB40 virus with spread of TB40-UL131Astop virus in PDGFR-α-silenced HFF.

To evaluate the role of PDGFR-α in cell-associated spread, we first performed experiments with non-transfected HFF. Confluent monolayers of HFF were infected with wt TB40 at a very low m.o.i and infected cells in discrete foci were counted. Addition of a methylcellulose overlay, which impedes spread via supernatant virus, and neutralizing anti-gB and anti-gH antibodies significantly reduced the sizes of foci but did not abolish their formation (Fig 6a). Similarly, addition of PDGFR-α-Fc reduced focal spread (Fig 6a). PDGFR-α-Fc equally inhibited infections of HFF with wt TB40 and TB40-UL131Astop viruses which excludes that PDGFR-α-Fc-resistant spread just reflects spread driven by the pentameric complex (Fig 6b). It is of note that spread of TB40-ΔgO virus, which in HFF cultures is less efficient than spread of wt TB40 virus (S8a Fig, [15]), can also be decreased by methylcellulose and also is resistant to neutralizing antibodies (S8a Fig). However, an anti-UL131A antiserum could completely block cell spread (focus size = 1) (S8a Fig). This extraordinary neutralization indicates that spread of TB40-ΔgO virus is completely dependent on the pentameric complex. Interestingly, in HUVEC cultures, in which spread of TB40-ΔgO virus is more efficient, the anti-UL131A antiserum could not completely block focal spread (S8b Fig).

To find out whether the PDGFR-α-Fc-resistant spread of wt TB40 and TB40-UL131Astop viruses reflects a PDGFR-α-independent spread mode or not, we repeated the spread experiments using HFF transfected with pooled PDGFR-α siRNAs or as a control non-targeting (NT) siRNAs. The experiments were all performed in the presence of methylcellulose to simultaneously block spread via supernatant virus. Virus spread of wt TB40 and TB40-UL131Astop viruses was clearly reduced, albeit to significantly different levels (Fig 6c and S9a Fig). While spread of TB40-UL131Astop virus was virtually abolished, spread of wt TB40 virus (Fig 6c) was strongly reduced, but not completely abolished. For comparison, spread of the HCMV strain VR1814, which, like wt TB40, also expresses both gH/gL complexes, was similarly inhibited in PDGFR-α-silenced HFF (S9b Fig). Spread of TB40-ΔgO virus was not affected by cellular PDGFR-α levels, but could be completely inhibited (focus size = 1) by anti-UL131A antibodies (Fig 6c). The residual spread of wt TB40 virus in PDGFR-α-knockdown HFF was in the same range as spread of TB40-ΔgO virus in PDGFR-α-knockdown HFF and very likely represents gH/gL/UL128,130,131A-dependent focal spread. In summary, using PDGFR-α-knockdown cells and comparing gO-positive and gO-negative viruses, we could show that cell-associated HCMV spread in HFF cultures is either driven by gH/gL/gO binding to PDGFR-α or by the gH/gL/UL128,130,131A complex but not by other independent pathways.

## Discussion

Expression of PDGFR-α is important for infection of cells with HCMV. Knockdown of PDGFR-α, soluble PDGFR-α, anti-PDGFR-α antibodies or PDGF-AA all strongly inhibit HCMV infection and re-introduction of PDGFR-α in knockout cells reconstitutes infection [23,32]. Using recombinant HCMV gB, binding of gB to PDGFR-α has been shown [23,42]. Similarly, recombinant gH/gL/gO complex has been used to precipitate PDGFR-α from cell lysates [32]. In the latter study, a PDGFR-α—gH/gL/gO complex with PDGFR-α directly interacting with gO could be visualized by electron microscopy. Additionally, all three studies proposed an enhancement of infection by activation of PDGFR-α [23,32,42]. Thus, PDGFR-α as an entry receptor for HCMV recognized by gB and/or gH/gL/gO and PDGFR-α as a signaling ligand for HCMV are two concepts which require confirmation or disproof.

We analyzed the interaction of the HCMV envelope gH/gL/gO complex with cell surface proteins using virus particles bound to the surface of fibroblasts and could confirm the interaction of gH/gL/gO with PDGFR-α. We found that PDGFR-α interacted with gH, gO and gB. By using an HCMV mutant lacking gO, we could sort out that the gH/gL/gO complex is the binding partner for PDGFR-α. An interaction of gB and PDGFR-α could only be observed when gH/gL/gO is formed. This suggests that gB either binds to PDGFR-α after PDGFR-α has formed a complex with gH/gL/gO or that a preformed gB—gH/gL/gO complex binds to PDGFR-α. gH/gL—gB interactions have been described before in cells co-expressing recombinant gB and gH/gL [7] and very recently also in HCMV virions [35]. The latter publication showed that gB does not interact with gH/gL/gO complexes in virions, [35] which would suggest a scenario in which a PDGFR-α—gH/gL/gO complex recruits gB to form a functional fusion complex [7]. Our co-precipitation experiments confirm the interaction of recombinant gH/gL/gO with PDGFR-α [32], but not a gH/gL/gO-independent interaction of gB and PDGFR-α [23]. The direct interaction of recombinant gB and PDGFR-α in cells over-expressing gB might reflect an over-expression phenomenon [23,42]. In summary, the binding studies by Kabanova et al. [32] and our co-precipitation experiments support the concept that PDGFR-α is an entry receptor for HCMV recognized by gH/gL/gO.

We also addressed the aspect of activation of PDGFR-α by HCMV. The first report proposing PDGFR-α as a co-receptor for HCMV entry strongly focused on PDGFR-α activation by HCMV [23]. It described HCMV-dependent phosphorylation of PDGFR-α and downstream Akt and inhibition of this activation by the protein kinase inhibitor imatinib mesylate or anti-PDGFR-α antibodies. In the same line, Kabanova et al. [32] claimed that binding of gH/gL/gO to fibroblasts results in tyrosine phosphorylation of PDGFR-α. We could not confirm these findings. As PDGFR-α and Akt phosphorylations can also be induced by FCS, we strictly controlled that both mock treatment and HCMV preparations contained equal amounts of FCS. We looked for activation of PDGFR-α after binding of HCMV to HFF and MRC-5 fibroblasts and neither observed phosphorylation of PDGFR-α nor of downstream Akt, although we used an m.o.i. of 10 for infection. Additionally, we could not inhibit infection of HFF with the protein kinase inhibitor imatinib mesylate. As Soroceanu et al. [23] showed phosphorylation of PDGFR-α after infection of HELF, the observed discrepancy might be due to differences in PDGFR-α-activation patterns between HFF and HELF [38]. By comparing infection of 293 cells overexpressing either full-length PDGFR-α or a signaling-incompetent truncated PDGFR-α protein, we could show that PDGFR-α-dependent enhancement of infection is independent of activation of PDGFR-α. Thus, gH/gL/gO-driven entry, although it may be accompanied by activation of PDGFR-α in certain cell types, is not dependent on activation of PDGFR-α. This identifies PDGFR-α rather as a fusion-triggering entry receptor than as a signaling ligand enhancing HCMV infection.

It has repeatedly been shown that effective infection of fibroblasts is dependent on the gH/gL/gO complex [14–16,21]. By using gO-negative HCMV, we could unequivocally show that gH/gL/gO-dependent and PDGFR-α-dependent infection are congruent. We could efficiently block infection of fibroblasts by pre-incubating virions with PDGFR-α-Fc fusion protein and by pre-incubating cells with the PDGFR-α ligand PDGF-AA when virions were gO-positive, but not when gO was knocked out and infection was dependent on the pentameric gH/gL/UL128,130,131A complex [14,15]. Importantly, inhibition of wt TB40 virus by co-incubation of virions with recombinant PDGFR-α-Fc protein reduced the infectivity for fibroblasts about 500 fold. When we normalized infectivity of wt TB40 and TB40-ΔgO virus preparations for their MCP content, we observed a difference in infectivity in the same range. This indicates that inhibition of wt HCMV virions with PDGFR-α-Fc mirrors the phenotype of a genetically gO-negative mutant. This finding and our experiments showing (i) neutralization of an HCMV ΔgO mutant by anti-gB, anti-gH, and anti-UL131A antibodies and (ii) that, in contrast to spread in HFF cultures, ΔgO virus spread in HUVEC cultures is more efficient than spread of wildtype virus strongly support that virus particles lacking gO are not just defect particles with a residual undefined infectivity, but rather virus particles whose infectivity is restricted to gH/gL/UL128,130,131A-driven entry exerted in concert with gB. This makes them a valuable tool to study for example gH/gL/UL128,130,131A-driven entry.

The PDGFR-α—gH/gL/gO interaction seems to start the predominant entry pathway for infection of cells with free virus, provided the cells abundantly express PDGFR-α on their surface. Different HCMV strains incorporate different relative amounts of the gH/gL complexes into their virions [16,17,43,44] which makes it difficult to evaluate the contribution of each complex to entry by comparing different strains [32]. Yet, independently of the HCMV strain used, deletion of gO massively reduces infectivity of cell-free virus [14–17,45]. Thus, independent of the HCMV strain, gH/gL/gO very likely is the predominant driver of infection with cell-free virus. In line with findings from Vanarsdall et al. [37], we could show that over-expression of PDGFR-α strongly increases the susceptibility of cells to infection, both in the absence and in the presence of the pentameric complex. As infection with TB40-ΔgO virus was not affected by overexpression of PDGFR-α, we can conclude that the PDGFR-α-dependent enhancement is gH/gL/gO-dependent.

Cell-associated virus spread is based on coordinated changes in cell architecture and cell surfaces to efficiently transfer virus to neighboring cells [39,46]. There are reports suggesting that cell-associated HCMV spread may be totally independent of virion glycoproteins or dependent on glycoproteins different from those promoting cell-free HCMV spread [40,41]. This was supported by findings that cell-associated spread in cell culture cannot be inhibited by neutralizing antibodies directed against virion proteins know to be involved in cell-free virus spread [47]. Similarly, by using recombinant PDGFR-α-Fc, we could not reduce spread of wt TB40 or TB40-UL131Astop viruses to a level below the level achieved by methylcellulose overlays known to block spread of supernatant virus. Yet, when we silenced PDGFR-α in HFF, cell-associated spread of TB40-UL131Astop virus was abolished and cell-associated spread of wt TB40 virus was reduced to the level observed for TB40 virus lacking gO. This indicates that virus spread in cell culture, independently of whether it can be inhibited by antibodies or not, is either driven by the gH/gL/gO—PDGFR-α interaction or by gH/gL/UL128.130,131A. The relative contributions of gH/gL/gO and gH/gL/UL128,130,131A very likely depend on the HCMV strain or isolate. They very likely also vary with the relative amounts of these complexes in virions and the relative amounts of PDGFR-α and the receptor(s) recognized by the pentameric complex on host cell surfaces. Our finding that TB40-ΔgO virus spread can be completely inhibited by anti-UL131A antibodies in HFF cultures, but not in HUVEC cultures additionally suggests that the failure of antibodies to block spread is rather due to local high concentrations of receptors or their ligands than to a failure of antibodies to access their target proteins.

According to our data, infection of epithelial and endothelial cells which express no or undetectable levels of PDGFR-α should be equally susceptible to infection with virions expressing no, low or high amounts of gH/gL/gO. Yet, it has been shown that particles containing both gH/gL complexes show a higher capacity to infect endothelial or epithelial cells than particles containing only the pentameric complex [14,17]. Our findings described here cannot explain this. We can only speculate that the gH/gL/gO complex, by promoting PDGFR-α-independent attachment to cell surfaces, may enhance binding of virus particles and thus increase the chance for a subsequent interaction between gH/gL/UL128,130,131A and its cellular receptor. Our hypothesis would follow a proposal of Zhou et al. [17] who suggested an entry enhancement by gH/gL/gO for epithelial cells which is independent of the interaction between the gH/gL/UL128,130,131A complex and its receptor. In the same line, Kabanova et al. [48] observed that epithelial cell infection could be blocked with antibodies directed against gO although they do not express detectable levels of PDGFR-α receptor.

To summarize our findings, we believe that we could confirm that PDGFR-α is the cellular receptor for the HCMV gH/gL/gO complex. Our findings very well fit prominent phenotypes found in cell culture for gH/gL-associated mutations, for example the strongly reduced infection of fibroblasts by mutants expressing less gO or lacking gO [14–16,19], the inability of laboratory strains, which have lost the ability to form the pentameric complex, to infect cell types with no or low levels of PDGFR-α [24,37], and the susceptibility of HCMV infection to cellular PDGFR-α levels [24,37,42,49,50].

Consequently, for HCMV expressing high levels of gH/gL/gO, the PDGFR-α level of target cells is the major factor determining cell tropism. Only if cells express no or low levels of PDGFR-α, entry pathways dependent on the gH/gL/UL128,130,131A complex will become important. This finding is of importance when the capacity of HCMV to infect cells in culture is used to characterize the HCMV cell tropism. Culture conditions [51,52] and also transforming antigens [24] may down- or up-regulate PDGFR-α and thus affect the susceptibility of cells to HCMV infection. HCMV tropism defined in cell culture may thus be misleading. The role of PDGFR-α-driven susceptibilities to infection should also be kept in mind when discussing the role of HCMV in glioblastoma [53] and arteriosclerosis [54], two disease conditions associated with tissues which express high levels of PDGFR-α.

In MCMV infections of the mouse, gH/gL/gO has been shown to play a central role in efficient infection of first target cells [18]. Transferred to HCMV infections, the gH/gL/gO—PDGFR-α interaction may thus determine which cells and how efficiently they are initially infected. We suggest therefore that PDGFR-α—gH/gL/gO interactions might be crucial for horizontal transmission via free virus from body fluids like urine or breast milk and perhaps also for vertical transmission of HCMV. This makes the PDGFR-α—gH/gL/gO interaction an interesting target for HCMV vaccines or antiviral therapies against HCMV.

## Materials and methods

### Cells

Primary human foreskin fibroblasts (HFF) (PromoCell, Germany), passage 7–23, human fetal lung fibroblasts (MRC-5 cells; ATCC-CCL-171) and human embryonic kidney cells (293 cells; ATCC-CRL-1573; [55]) were maintained in Dulbecco’s modified Eagle’s medium (DMEM) supplemented with 10% fetal calf serum (FCS). Primary human umbilical vein endothelial cells (HUVEC) (LONZA, USA), passage 1–6, were maintained in an EGM-2 MV BulletKit medium system (LONZA, USA). Human retinal pigment epithelial cells (ARPE-19; ATCC-CRL-2302) were maintained in DMEM F12 supplemented with 10% FCS.

### Antibodies, recombinant proteins and inhibitors

For co-precipitation and competition experiments, human PDGFR-α-Fc and human PDGFR-β-Fc fusion proteins (R&D Systems) and recombinant human PDGF-AA and human PDGF-BB (R&D Systems) were used. For inhibition of PDGFR-α phosphorylation, imatinib mesylate (Sigma) was used. Antibodies specific for HCMV proteins were mouse anti-gH (14-4b and AP86-SA4), human anti-gB (SM5-1 [56]), mouse anti-MCP (all kindly provided by M. Mach, University Erlangen-Nürnberg, Germany), mouse anti-gO.02 [57], mouse anti-UL128 (4B10, kindly provided by T. Shenk, University of Princeton, USA), mouse anti-gB (2F12; Abcam), mouse anti-HCMV immediate early protein 1 (IE1) (Perkin Elmer), rabbit anti-UL131A antiserum [12]. Antibodies specific for cellular proteins were rabbit anti-Akt (Cell Signaling), rabbit anti-Phospho-Akt (Ser 473) (Cell Signaling), mouse anti-PDGFR-α (C9; Santa Cruz Biotechnology), mouse anti-PDGFR-α (35248; R&D Systems), mouse anti-PDGFR-α (2D2-1A11, Sigma), rabbit anti-Phospho-PDGFR-α (Y742; R&D Systems), rabbit anti-Phospho-PDGFR-α (Y762; Cell Signaling), mouse anti-β actin (AC-74; Sigma), mouse anti-GAPDH (GA1R, Thermo Scientific). Detecting antibodies/reagents used were peroxidase-coupled goat anti-mouse (Sigma), peroxidase-coupled kappa chain-specific goat anti-mouse (Dianova), peroxidase-coupled goat anti-rabbit (Sigma), Fluor488-coupled goat anti-mouse antibody (Invitrogen) and streptavidin-peroxidase polymer (Sigma).

### Viruses and virus stock preparation

All viruses used were bacterial artificial chromosome (BAC)-derived HCMV: wt TB40 virus [58], TB40-UL131Astop virus (TB40 mutant carrying a stop codon after the first 6 aa) [20], TB40-ΔgO virus (TB40 mutant lacking the 533 N-terminal nucleotides) [15,20], wt TB40-luc virus [19], TB40-UL131Astop-luc virus [19], and TB40-ΔgO-luc virus [19]. For preparation of cell-free virus, supernatants of infected HFF showing a complete cytopathic effect were cleared of cellular debris at 3.000 g for 15 min and then virus pelleted at 26.000 g for 3 hours. Virus pellets were resuspended in DMEM containing 5% FCS and stored at -80°C. Titers of virus stocks were determined by a TCID_50_ assay performed on 96 well plates on HFF. To infect cells, medium was removed from 90% confluent cell monolayers and replaced by virus diluted in DMEM containing 5% FCS. In infection experiments using TB40-ΔgO, all virus infections were enhanced by a centrifugation step (30 min, 2.000 g at RT), followed by incubation at 37°C for 90 min.

### Cell surface biotinylation

HFF were biotinylated in 0.5 mg/ml EZ-Link Sulfo-NHS-LC-LC-Biotin (Thermo Scientific) in PBS for 30 min at RT. The reaction was stopped by washing the cells 3 times with 100 mM glycine in PBS.

### Immunoprecipitation

Cells or virus stocks were lysed in lysis buffer (20 mM TrisHCl (pH 8.0), 150 mM NaCl and 1% Triton; phosphatase inhibitor cocktail (cOmplete mini; Roche)). Lysates were precleared with protein A sepharose beads (GE Healthcare) and co-incubated with antibodies or Fc fusion proteins at 4°C overnight. Then protein-antibody complexes were precipitated with protein A sepharose beads.

The precipitates were dissociated in sample buffer (0.13 M Tris-HCl (pH 6.8), 6% SDS, 10% a-thioglycerol) and subjected to SDS-polyacrylamide gel electrophoresis (SDS-PAGE), followed by either silver stain (FireSilver Staining Kit, Proteome factory, Berlin) or Western blot analysis using nitrocellulose membranes (GE Healthcare) for protein transfer and Super Signal West Pico chemiluminescence substrate (ThermoScientific) for detection.

### Mass spectrometry

Proteins in gel slices from silver stained gels were identified by high resolution nanoHPLC-ESI-MSMS chromatography (Proteome Factory, Berlin, Germany) and searched against human cellular proteins.

### Cloning, expression and purification of recombinant HCMV gO-mIgG2bFc fusion protein

The open reading frame of gO derived from HCMV strain TB40-BAC4 [59] lacking the N-terminal signal peptide (aa 1–28) was cloned into the pFUSE-mIgG2BFc2 vector (Invivogen). 293 cells were transfected with pFUSE-gO-mIgG2BFc2 using polyethylenimine. Three hours after transfection medium was exchanged for OptiPRO serum-free medium (LifeTechnologies) and supernatants were harvested 96 hours after transfection. Total supernatant proteins were precipitated with ethanol/125 mM NaCl to control expression by Western blot analysis. Fc proteins were purified from supernatants by precipitation with protein A sepharose (GE Healthcare) followed by elution using ImmunoPure IgG elution buffer, pH 2.8 (Thermo Scientific). Fc protein amounts were determined by a capture ELISA on NeutrAvidin coated plates (Thermo Scientific) using a biotinylated goat anti-mouse IgG (Fc-specific) antibody (Sigma) to capture Fc proteins and a peroxidase-conjugated goat anti-mouse IgG (Fc-specific) antibody (Sigma) and TMB substrate (BD Biosciences) to detect Fc proteins.

### Indirect immunofluorescence

For indirect immunofluorescence, adherent cells were fixed in 50% acetone/50% methanol and stained using anti-IE1 antibody and Fluor488-coupled anti-mouse antibody. For counterstaining of cell nuclei, cells were incubated in PBS containing 5 mg ml^-1^ Hoechst 333258 (Invitrogen).

### FACS staining

To detect surface expression of PDGFR-α or to detect cell surface binding of gO-Fc fusion proteins, HFF, HUVEC or 293 cells were detached from plates using 0.5 mM Na-EDTA and co-incubated with anti-PDGFR-α antibodies or Fc proteins, respectively. PDGFR-α or binding of Fc proteins was detected with Fluor 488-coupled anti-mouse antibodies and analyzed on a BD FACS Canto II cytometer using BD FACS Diva software (BD Biosciences).

### Luciferase assay

HFF or 293 cells were grown in 96 well plates (20,000 cells/well) and infected in triplicates for 90 min. Inocula were then replaced by medium supplemented with 300 μg ml^-1^ phosphono acetic acid (PAA) to block viral replication (Sigma). 48 hours after infection cells were lysed in 50 μl lysis buffer (25 mM Tris/H_3_PO_4_, 2 mM EDTA, 2 mM DTT, 10% glycerol, 5% Triton-X 100) and luciferase activity was determined for 20 μl of lysate with a luciferase assay system (PromoCell) according to the manufacturer’s instructions.

### Infection of 293 cells over-expressing PDGFR-α

293 cells were transfected with pCMV-human PDGFR-α (Sino Biological Inc.) or pCMV-PDGFR-α(1–558) using Fugene (Promega). 24 hours after transfection cells were seeded in 96 well plates and 24 hours later infected with luciferase-expressing HCMV.

### In vitro mutagenesis

pCMV-PDGFR-α(1–558) was cloned by introducing a stop codon at position 559 into pCMV-PDGFR-α using the QuikChange II XL Site-Directed Mutagenesis Kit (Agilent Technologies) according to the manufacturer’s instructions (mutagenesis primers: forward 5’-TGATTGATTCAATGACCCTTCAGCGAATTTCATACCTCG-3’ and reverse 5’-CGAGGTATGAAATTCGCTGAAGGGTCATTGAATCAATCA-3’).

### Silencing of PDGFR-α using siRNAs

HFF were transfected in 12 well plates (80.000 cells per well) with 50 nM siGENOME human PDGFR-α (5156) siRNA SMARTpool (Dharmacon) or siGENOME Non-Targeting siRNA Pool #2 (Dharmacon) using RNAiMAX (Fischer Scientific) as transfection reagent.

## Supporting information

S1 Fig Anti-gH antibodies co-precipitate a cell surface protein from lysates of HFF co-incubated with TB40-UL131Astop virions. HFF surface proteins were biotinylated and lysates of HFF (1), lysates of HFF mixed with lysates of TB40-UL131Astop virions (2) and lysates of HFF co-incubated with TB40-UL131stop virions (3) were subjected to anti-gH immunoprecipitation. Proteins were separated by SDS-PAGE followed by transfer to nitrocellulose membranes or a silver stain. Biotinylated proteins were detected using streptavidin-peroxidase polymer reagent. The positions of co-precipitated biotinylated proteins at about 180 kDa (left panel) and the respective bands in the silver gel (right panel) are indicated by arrows. The positions of gH and co-precipitated gO are indicated. The gel slices cut out for mass spectrometry are indicated by red boxes.
(TIF)

S2 Fig wt TB40 virion gH differs from TB40-ΔgO virion gH. Lysates of HFF infected with wt TB40 or TB40-ΔgO virus and lysates of respective cell-free virions were analyzed by Western blot using an anti-gH antibody.
(TIF)

S3 Fig Binding of gO-Fc fusion protein to HFF cell surfaces. (a) Total cell extracts of HFF, MRC-5, 293 cells, ARPE-19, and HUVEC were analyzed by Western blot for the expression of PDGFR-α and β-actin. (b) 293 cells were transfected with pFUSE-mIgG2B-Fc (1) or pFUSE-gO-mIgG2B-Fc (2). 96 hours after transfection, cell culture supernatants were collected and proteins precipitated with ethanol. Fc fusion proteins were detected by Western blot analysis using a peroxidase-coupled anti-mouse antibody. (c) HFF and HUVEC were co-incubated with equal amounts of purified gO-Fc fusion protein or as a control Fc fusion protein and binding determined by FACS analysis using a Fluor 488-labelled goat anti-mouse IgG antibody.
(TIF)

S4 Fig Infection of HFF with wt TB40 and TB40-ΔgO viruses. (a) Neutralization of infection with antibodies and (b,c) direct comparison of two methods to quantify HCMV infection of HFF: indirect immunofluorescence staining for HCMV IE1 (b) and luciferase assay (c). (a,b,c) HFF were infected on 96 well plates. Before infection, wt TB40 and TB40-ΔgO viruses were pre-incubated with (a) anti-gB antibodies (SM5-1, 2 μg ml^-1^), anti-gH antibodies (14-4b), anti-UL131A rabbit antiserum (1:40), control rabbit antiserum (1:40) or medium (mock-treated) or (b,c) PDGFR-α-Fc (300 ng ml^-1^) or medium (mock-treated) for one hour at 4°C. wt and ΔgO virus inocula were adjusted to result in comparable numbers of infected HFF under mock conditions. Cells were infected using centrifugal enhancement, washed three times with PBS after infection and then incubated for 24 hours before infection was detected by indirect immunofluorescence staining for HCMV IE1 (a,b) or by a luciferase assay (c). Under (b) the percentages of IE1-positive nuclei are indicated. Shown are means +/- SD of representative experiments done in triplicates.
(TIF)

S5 Fig Abundant TB40-ΔgO-luc particles do not interfere with inhibition of wt TB40-luc virus by PDGFR-α-Fc. HFF were infected on 96 well plates. Before infection, TB40-UL131Astop-luc or TB40-ΔgO-luc virus or a mixture of both viruses were pre-incubated with 3 μg ml^-1^ PDGFR-α-Fc or as a control medium for one hour at 4°C. Cells were infected with the virion—PDGFR-Fc mixtures using centrifugal enhancement, washed three times with PBS, and then incubated for 24 hours before infection was determined by a luciferase assay. Shown are means +/- SD of one representative experiment done in triplicates. Inhibition of TB40-UL131Astop-luc by PDGFR-α-Fc was below the detection limit.
(TIF)

S6 Fig Overexpression of PDGFR-α in 293 cells. 293 cells were transfected with pCMV-PDGFR-α or a control vector. (a) Total cell extracts were analyzed for PDGFR-α expression by Western blot 24 and 48 hours after transfection. (b) 24 hours after transfection, cells were either stained by Hoechst 33258 to visualize nuclei or by indirect immunofluorescence using a PDGFR-α-specific antibody. (c) and (d) Transfected 293 cells were infected with wt TB40-luc and then incubated for 24 hours before infection was in parallel detected by indirect immunofluorescence staining for HCMV IE1 (c) or a luciferase assay (d). Under (c) the percentages of IE1-positive nuclei are indicated. Shown are means +/- SD of one representative experiment done in triplicates.
(TIF)

S7 Fig Overexpression of truncated PDGFR-α in 293 cells. (a) Schematic presentation of full-length and truncated PDGFR-α. The open reading frame of full-length PDGFR-α consists of 1089 amino acids. The last amino acid of truncated PDGFR-α is Arg558. The transmembrane domain ranges from Ala529 to Trp549. (b) 293 cells were transfected with pCMV-PDGFR-α, pCMV-PDGFR-α(1–558), or a vector control. 24 hours after transfection, cell surfaces of transfected cells were stained with an anti-PDGFR-α antibody (35248) or an isotype control and a secondary Fluor 488-labelled anti-mouse antibody and analyzed by FACS.
(TIF)

S8 Fig Characterization of TB40-ΔgO virus spread in cell culture. (a) Confluent monolayers of HFF were infected with TB40-ΔgO virus at a very low m.o.i. After infection, cells were either overlaid with methylcellulose or medium containing anti-gB antibodies (SM5-1, 2μg ml^-1^), anti-gH antibodies (14-4B), anti-UL131A antiserum (1:40), control rabbit antiserum (1:40), or no inhibitor (mock-treated). 5 days later, cells were stained for HCMV IE1 by indirect immunofluorescence and cells per focus counted. For each treatment, at least 12 foci were counted and depicted as means +/- SD. Shown is one representative experiment. Asterisks represent P<0.001 values determined by comparing foci in mock-treated monolayers with foci in monolayers overlaid with methylcellulose or co-incubated with antibodies (Mann-Whitney Rank Sum test). (b) Confluent monolayers of HUVEC were infected with wt TB40 or TB40-ΔgO virus at a very low m.o.i. After infection, cells were either incubated with medium containing anti-UL131A antiserum (1:40) or medium alone (mock-treated). Foci were analyzed as described under (a). P values were determined by comparing foci of wt TB40 and TB40-ΔgO virus infections either under mock conditions or in the presence of anti-UL131A antibodies (Mann-Whitney Rank Sum test).
(TIF)

S9 Fig Silencing of PDGFR-α reduces cell-associated spread of gH/gL/gO-positive HCMV in fibroblast cultures. NT siRNA- or PDGFR-α siRNA-transfected HFF 48 hours after transfection were mixed with HFF infected with (a) wt TB40, TB40-UL131Astop or TB40-ΔgO virus or b) VR1814. After adherence, cells were overlaid with methylcellulose. 5 days later, cells were stained for HCMV IE1 by indirect immunofluorescence. (a) Representative stainings of the analysis shown in Fig 6c. (b) One representative experiment for which at least 20 foci were counted and depicted as means +/- SD.
(TIF)

## References

[1] Boppana SB, Britt WJ. Synopsis of clinical aspects of human cytomegalovirus disease In: Reddehase MJ, editors. Cytomegaloviruses: From Molecular Pathogenesis to Intervention. Norfolk, UK: Caister Academic Press 2013; pp 1–25.

[2] Plachter B, Sinzger C, Jahn G. Cell types involved in replication and distribution of human cytomegalovirus. Adv Virus Res. 1996; 46:195–261. 8824701 10.1016/s0065-3527(08)60073-1

[3] Compton T, Nowlin DM, Cooper NR. Initiation of human cytomegalovirus infection requires initial interaction with cell surface heparan sulfate. Virology. 1993; 193:834–841. 10.1006/viro.1993.1192 8384757

[4] Boyle KA, Compton T. Receptor-binding properties of a soluble form of human cytomegalovirus glycoprotein B. J Virol. 1998; 72:1826–1833. 9499033 10.1128/jvi.72.3.1826-1833.1998 PMC109472

[5] Kari B, Gehrz R. A human cytomegalovirus glycoprotein complex designated gC-II is a major heparin-binding component of the envelope. J Virol. 1992; 66:1761–1764. 1310777 10.1128/jvi.66.3.1761-1764.1992 PMC240930

[6] Heldwein EE. gH/gL supercomplexes at early stages of herpesvirus entry. Curr Opin Virol. 2016; 18:1–8. 10.1016/j.coviro.2016.01.010 26849495 PMC4970976

[7] Vanarsdall AL, Ryckman BJ, Chase MC, Johnson DC. Human cytomegalovirus glycoproteins gB and gH/gL mediate epithelial cell-cell fusion when expressed either in cis or in trans. J Virol. 2008; 82:11837–11850. 10.1128/JVI.01623-08 18815310 PMC2583677

[8] Hahn G, Revello MG, Patrone M, Percivalle E, Campanini G, Sarasini A et al Human cytomegalovirus UL131-128 genes are indispensable for virus growth in endothelial cells and virus transfer to leukocytes. J Virol. 2004; 78:10023–10033. 10.1128/JVI.78.18.10023-10033.2004 15331735 PMC515016

[9] Gerna G, Percivalle E, Lilleri D, Lozza L, Fornara C, Hahn G et al Dendritic-cell infection by human cytomegalovirus is restricted to strains carrying functional UL131-128 genes and mediates efficient viral antigen presentation to CD8+ T cells. J Gen Virol. 2005; 86:275–284. 10.1099/vir.0.80474-0 15659746

[10] Wang D, Shenk T. Human cytomegalovirus UL131 open reading frame is required for epithelial cell tropism. J Virol. 2005; 79:10330–10338. 10.1128/JVI.79.16.10330-10338.2005 16051825 PMC1182637

[11] Wang D, Shenk T. Human cytomegalovirus virion protein complex required for epithelial and endothelial cell tropism. Proc Natl Acad Sci U S A. 2005; 102:18153–18158. 10.1073/pnas.0509201102 16319222 PMC1312424

[12] Adler B, Scrivano L, Ruzcics Z, Rupp B, Sinzger C, Koszinowski U. Role of human cytomegalovirus UL131A in cell type-specific virus entry and release. J Gen Virol. 2006; 87:2451–2460. 10.1099/vir.0.81921-0 16894182

[13] Frascaroli G, Varani S, Moepps B, Sinzger C, Landini MP, Mertens T. Human cytomegalovirus subverts the functions of monocytes, impairing chemokine-mediated migration and leukocyte recruitment. J Virol. 2006; 80:7578–7589. 10.1128/JVI.02421-05 16840337 PMC1563711

[14] Wille PT, Knoche AJ, Nelson JA, Jarvis MA, Johnson DC. An HCMV gO-null mutant fails to incorporate gH/gL into the virion envelope and is unable to enter fibroblasts, epithelial, and endothelial cells. J Virol. 2009; 84:2585–2596. 10.1128/JVI.02249-09 20032184 PMC2820920

[15] Jiang XJ, Adler B, Sampaio KL, Digel M, Jahn G, Ettischer N et al UL74 of human cytomegalovirus contributes to virus release by promoting secondary envelopment of virions. J Virol. 2008; 82:2802–2812. 10.1128/JVI.01550-07 18184717 PMC2259017

[16] Li G, Nguyen CC, Ryckman BJ, Britt WJ, Kamil JP. A viral regulator of glycoprotein complexes contributes to human cytomegalovirus cell tropism. Proc Natl Acad Sci U S A. 2015; 112:4471–4476. 10.1073/pnas.1419875112 25831500 PMC4394275

[17] Zhou M, Lanchy JM, Ryckman BJ. Human cytomegalovirus gH/gL/gO promotes the fusion step of entry into all cell types whereas gH/gL/UL128-131 broadens virus tropism through a distinct mechanism. J Virol. 2015; 89:8999–9009. 10.1128/JVI.01325-15 26085146 PMC4524070

[18] Lemmermann NA, Krmpotic A, Podlech J, Brizic I, Prager A, Adler H et al Non-redundant and redundant roles of cytomegalovirus gH/gL complexes in host organ entry and intra-tissue spread. PLoS Pathog. 2015; 11:e1004640 10.1371/journal.ppat.1004640 25659098 PMC4450070

[19] Scrivano L, Sinzger C, Nitschko H, Koszinowski UH, Adler B. HCMV spread and cell tropism are determined by distinct virus populations. PLoS Pathog. 2011; 7:e1001256 10.1371/journal.ppat.1001256 21249233 PMC3020925

[20] Scrivano L, Esterlechner J, Muhlbach H, Ettischer N, Hagen C, Grunewald K et al The m74 gene product of murine cytomegalovirus (MCMV) is a functional homolog of human CMV gO and determines the entry pathway of MCMV. J Virol. 2010; 84:4469–4480. 10.1128/JVI.02441-09 20181688 PMC2863770

[21] Vanarsdall AL, Chase MC, Johnson DC. Human cytomegalovirus glycoprotein gO complexes with gH/gL, promoting interference with viral entry into human fibroblasts but not entry into epithelial cells. J Virol. 2011; 85:11638–11645. 10.1128/JVI.05659-11 21880752 PMC3209304

[22] Ryckman BJ, Chase MC, Johnson DC. HCMV gH/gL/UL128-131 interferes with virus entry into epithelial cells: evidence for cell type-specific receptors. Proc Natl Acad Sci U S A. 2008; 105:14118–14123. 10.1073/pnas.0804365105 18768787 PMC2544588

[23] Soroceanu L, Akhavan A, Cobbs CS. Platelet-derived growth factor-alpha receptor activation is required for human cytomegalovirus infection. Nature. 2008; 455:391–395. 10.1038/nature07209 18701889

[24] Xu S, Schafer X, Munger J. Expression of Oncogenic Alleles Induces Multiple Blocks to Human Cytomegalovirus Infection. J Virol. 2016; 90:4346–4356. 10.1128/JVI.00179-16 26889030 PMC4836323

[25] Li Q, Wilkie AR, Weller M, Liu X, Cohen JI. THY-1 Cell Surface Antigen (CD90) Has an Important Role in the Initial Stage of Human Cytomegalovirus Infection. PLoS Pathog. 2015; 11:e1004999 10.1371/journal.ppat.1004999 26147640 PMC4492587

[26] Wang X, Huong SM, Chiu ML, Raab-Traub N, Huang ES. Epidermal growth factor receptor is a cellular receptor for human cytomegalovirus. Nature. 2003; 424:456–461. 10.1038/nature01818 12879076

[27] Wang X, Huang DY, Huong SM, Huang ES. Integrin alphavbeta3 is a coreceptor for human cytomegalovirus. Nat Med. 2005; 11:515–521. 10.1038/nm1236 15834425 PMC1904494

[28] Feire AL, Koss H, Compton T. Cellular integrins function as entry receptors for human cytomegalovirus via a highly conserved disintegrin-like domain. Proc Natl Acad Sci U S A. 2004; 101:15470–15475. 10.1073/pnas.0406821101 15494436 PMC524452

[29] Nogalski MT, Chan GC, Stevenson EV, Collins-McMillen DK, Yurochko AD. The HCMV gH/gL/UL128-131 Complex Triggers the Specific Cellular Activation Required for Efficient Viral Internalization into Target Monocytes. PLoS Pathog. 2013; 9:e1003463 10.1371/journal.ppat.1003463 23853586 PMC3708883

[30] Bentz GL, Yurochko AD. Human CMV infection of endothelial cells induces an angiogenic response through viral binding to EGF receptor and beta1 and beta3 integrins. Proc Natl Acad Sci U S A. 2008; 105:5531–5536. 10.1073/pnas.0800037105 18375753 PMC2291133

[31] Feire AL, Roy RM, Manley K, Compton T. The glycoprotein B disintegrin-like domain binds beta 1 integrin to mediate cytomegalovirus entry. J Virol. 2010; 84:10026–10037. 10.1128/JVI.00710-10 20660204 PMC2937812

[32] Kabanova A, Marcandalli J, Zhou T, Bianchi S, Baxa U, Tsybovsky Y et al Platelet-derived growth factor-alpha receptor is the cellular receptor for human cytomegalovirus gHgLgO trimer. Nat Microbiol. 2016; 8:16082. 10.1038/nmicrobiol.2016.82 PMC4918640 27573107

[33] Huber MT, Compton T. The human cytomegalovirus UL74 gene encodes the third component of the glycoprotein H-glycoprotein L-containing envelope complex. J Virol. 1998; 72:8191–8197. 9733861 10.1128/jvi.72.10.8191-8197.1998 PMC110166

[34] Li L, Nelson JA, Britt WJ. Glycoprotein H-related complexes of human cytomegalovirus: identification of a third protein in the gCIII complex. J Virol. 1997; 71:3090–3097. 9060671 10.1128/jvi.71.4.3090-3097.1997 PMC191440

[35] Vanarsdall AL, Howard PW, Wisner TW, Johnson DC. Human Cytomegalovirus gH/gL Forms a Stable Complex with the Fusion Protein gB in Virions. PLoS Pathog. 2016; 12:e1005564 10.1371/journal.ppat.1005564 27082872 PMC4833381

[36] Buscher N, Paulus C, Nevels M, Tenzer S, Plachter B. The proteome of human cytomegalovirus virions and dense bodies is conserved across different strains. Med Microbiol Immunol. 2015; 204:285–293. 10.1007/s00430-015-0397-y 25732096

[37] Vanarsdall AL, Wisner TW, Lei H, Kazlauskas A, Johnson DC. PDGF receptor-alpha does not promote HCMV entry into epithelial and endothelial cells but increased quantities stimulate entry by an abnormal pathway. PLoS Pathog. 2012; 8:e1002905 10.1371/journal.ppat.1002905 23028311 PMC3441672

[38] Donovan J, Shiwen X, Norman J, Abraham D. Platelet-derived growth factor alpha and beta receptors have overlapping functional activities towards fibroblasts. Fibrogenesis Tissue Repair. 2013; 6:10–16. 10.1186/1755-1536-6-10 23663505 PMC3667071

[39] Sattentau Q. Avoiding the void: cell-to-cell spread of human viruses. Nat Rev Microbiol. 2008; 6:815–826. 10.1038/nrmicro1972 18923409

[40] Gerna G, Percivalle E, Baldanti F, Sozzani S, Lanzarini P, Genini E et al Human cytomegalovirus replicates abortively in polymorphonuclear leukocytes after transfer from infected endothelial cells via transient microfusion events. J Virol. 2000; 74:5629–5638. 10823870 10.1128/jvi.74.12.5629-5638.2000 PMC112050

[41] Silva MC, Schroer J, Shenk T. Human cytomegalovirus cell-to-cell spread in the absence of an essential assembly protein. Proc Natl Acad Sci U S A. 2005; 102:2081–2086. 10.1073/pnas.0409597102 15684067 PMC548577

[42] Cobbs C, Khan S, Matlaf L, McAllister S, Zider A, Yount G et al HCMV glycoprotein B is expressed in primary glioblastomas and enhances growth and invasiveness via PDGFR-alpha activation. Oncotarget. 2014; 5:1091–1100. 10.18632/oncotarget.1787 24658280 PMC4011586

[43] Murrell I, Tomasec P, Wilkie GS, Dargan DJ, Davison AJ, Stanton RJ. Impact of sequence variation in the UL128 locus on production of human cytomegalovirus in fibroblast and epithelial cells. J Virol. 2013; 87:10489–10500. 10.1128/JVI.01546-13 23885075 PMC3807394

[44] Zhou M, Yu Q, Wechsler A, Ryckman BJ. Comparative Analysis of gO Isoforms Reveals that Strains of Human Cytomegalovirus Differ in the Ratio of gH/gL/gO and gH/gL/UL128-131 in the Virion Envelope. J Virol. 2013; 87:9680–9690. 10.1128/JVI.01167-13 23804643 PMC3754086

[45] Ryckman BJ, Chase MC, Johnson DC. HCMV TR strain glycoprotein O acts as a chaperone promoting gH/gL incorporation into virions, but is not present in virions. J Virol. 2009; 84:2597–2609. 10.1128/JVI.02256-09 20032193 PMC2820934

[46] Mothes W, Sherer NM, Jin J, Zhong P. Virus cell-to-cell transmission. J Virol. 2010; 84:8360–8368. 10.1128/JVI.00443-10 20375157 PMC2918988

[47] Jacob CL, Lamorte L, Sepulveda E, Lorenz IC, Gauthier A, Franti M. Neutralizing antibodies are unable to inhibit direct viral cell-to-cell spread of human cytomegalovirus. Virology. 2013; 444:140–147. 10.1016/j.virol.2013.06.002 23849792

[48] Li G, Kamil JP. Viral Regulation of Cell Tropism in Human Cytomegalovirus. J Virol. 2015; 90:626–629. 10.1128/JVI.01500-15 26559829 PMC4702680

[49] Patrone M, Secchi M, Bonaparte E, Milanesi G, Gallina A. Cytomegalovirus UL131-128 products promote gB conformational transition and gB-gH interaction during entry into endothelial cells. J Virol. 2007; 81:11479–11488. 10.1128/JVI.00788-07 17686875 PMC2045554

[50] Berger AA, Gil Y, Panet A, Weisblum Y, Oiknine-Djian E, Gropp M et al Transition toward Human Cytomegalovirus Susceptibility in Early Human Embryonic Stem Cell-Derived Neural Precursors. J Virol. 2015; 89:11159–11164. 10.1128/JVI.01742-15 26292329 PMC4621109

[51] Marx M, Perlmutter RA, Madri JA. Modulation of platelet-derived growth factor receptor expression in microvascular endothelial cells during in vitro angiogenesis. J Clin Invest. 1994; 93:131–139. 10.1172/JCI116936 7506710 PMC293745

[52] Osornio-Vargas AR, Lindroos PM, Coin PG, Badgett A, Hernandez-Rodriguez NA, Bonner JC. Maximal PDGF-induced lung fibroblast chemotaxis requires PDGF receptor-alpha. Am J Physiol. 1996; 271:L93–L99. 8760137 10.1152/ajplung.1996.271.1.L93

[53] Cobbs CS. Cytomegalovirus and brain tumor: epidemiology, biology and therapeutic aspects. Curr Opin Oncol. 2013; 25:682–688. 10.1097/CCO.0000000000000005 24097102

[54] Popovic M, Smiljanic K, Dobutovic B, Syrovets T, Simmet T, Isenovic ER. Human cytomegalovirus infection and atherothrombosis. J Thromb Thrombolysis. 2012; 33:160–172. 10.1007/s11239-011-0662-x 22161772

[55] Graham FL, Smiley J, Russel WC, Nairn R. Characteristics of a human cell line transformed by DNA from human adenovirus type 5. J Gen Virol. 1977; 36:59–74. 10.1099/0022-1317-36-1-59 886304

[56] Potzsch S, Spindler N, Wiegers AK, Fisch T, Rucker P, Sticht H et al B cell repertoire analysis identifies new antigenic domains on glycoprotein B of human cytomegalovirus which are target of neutralizing antibodies. PLoS Pathog. 2011; 7:e1002172 10.1371/journal.ppat.1002172 21852946 PMC3154849

[57] Laib Sampaio K, Stegmann C, Brizic I, Adler B, Stanton RJ, Sinzger C. The contribution of pUL74 to growth of human cytomegalovirus is masked in the presence of RL13 and UL128 expression. J Gen Virol. 2016; 10. 10.1099/jgv.0.000475 PMC5156331 27050420

[58] May JS, de Lima BD, Colaco S, Stevenson PG. Intercellular gamma-herpesvirus dissemination involves co-ordinated intracellular membrane protein transport. Traffic. 2005; 6:780–793. 10.1111/j.1600-0854.2005.00316.x 16101681

[59] Sinzger C, Hahn G, Digel M, Katona R, Sampaio KL, Messerle M et al Cloning and sequencing of a highly productive, endotheliotropic virus strain derived from human cytomegalovirus TB40/E. J Gen Virol. 2008; 89:359–368. 10.1099/vir.0.83286-0 18198366

