# Supplementary figures and images for: Human cytomegalovirus glycoprotein complex gH/gL/gO uses PDGFR-α as a key for entry
Source: PLoS Pathog. 2017 Apr 12;13(4):e1006281. doi: 10.1371/journal.ppat.1006281 (PMC5389851; doi:10.1371/journal.ppat.1006281)

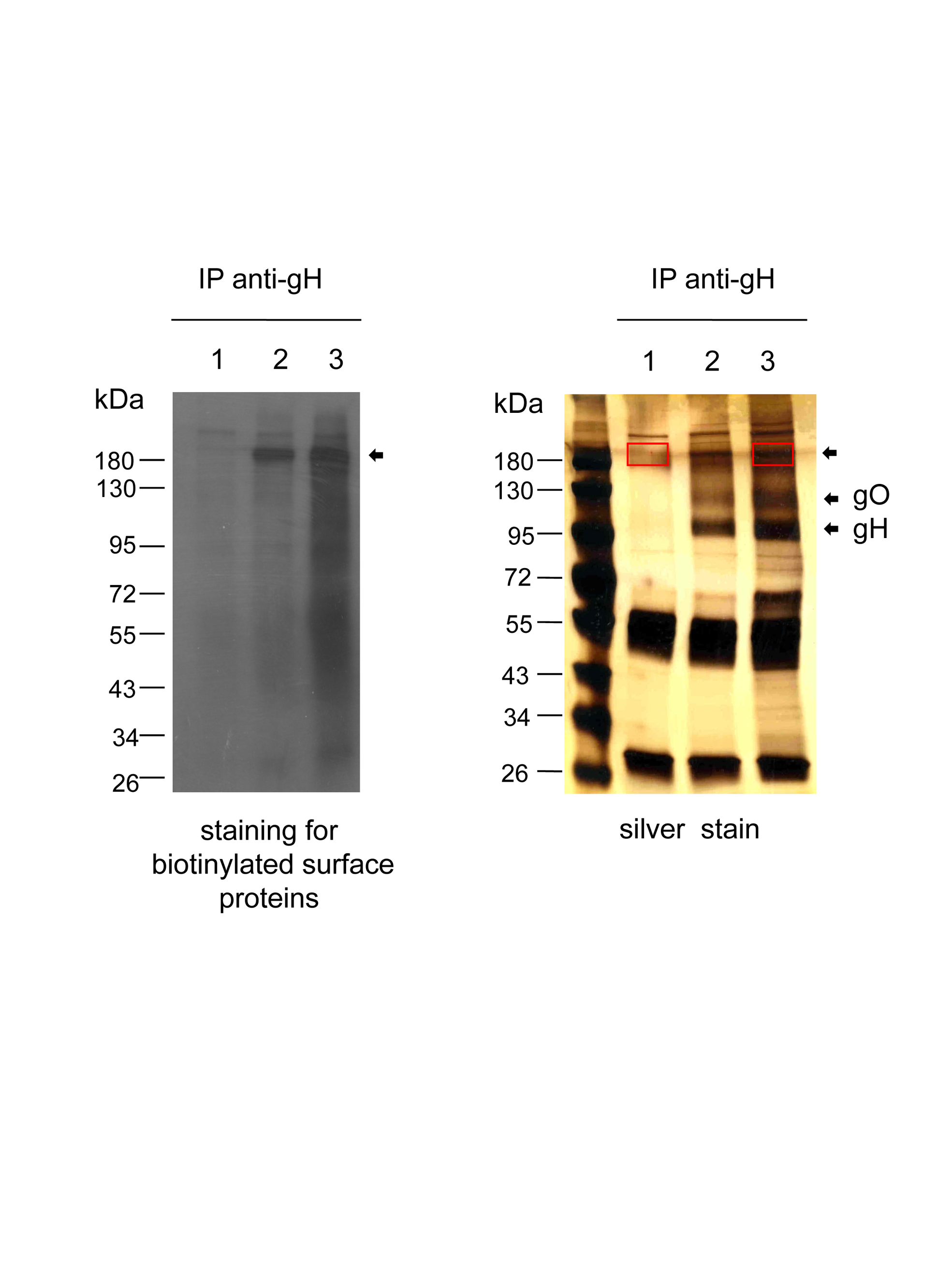

Supplement: S1 Fig — HFF surface proteins were biotinylated and lysates of HFF (1), lysates of HFF mixed with lysates of TB40-UL131Astop virions (2) and lysates of HFF co-incubated with TB40-UL131stop virions (3) were subjected to anti-gH immunoprecipitation. Proteins were separated by SDS-PAGE followed by transfer to nitrocellulose membranes or a silver stain. Biotinylated proteins were detected using streptavidin-peroxidase polymer reagent. The positions of co-precipitated biotinylated proteins at about 180 kDa (left panel) and the respective bands in the silver gel (right panel) are indicated by arrows. The positions of gH and co-precipitated gO are indicated. The gel slices cut out for mass spectrometry are indicated by red boxes. (TIF) [file ppat.1006281.s001.tif]

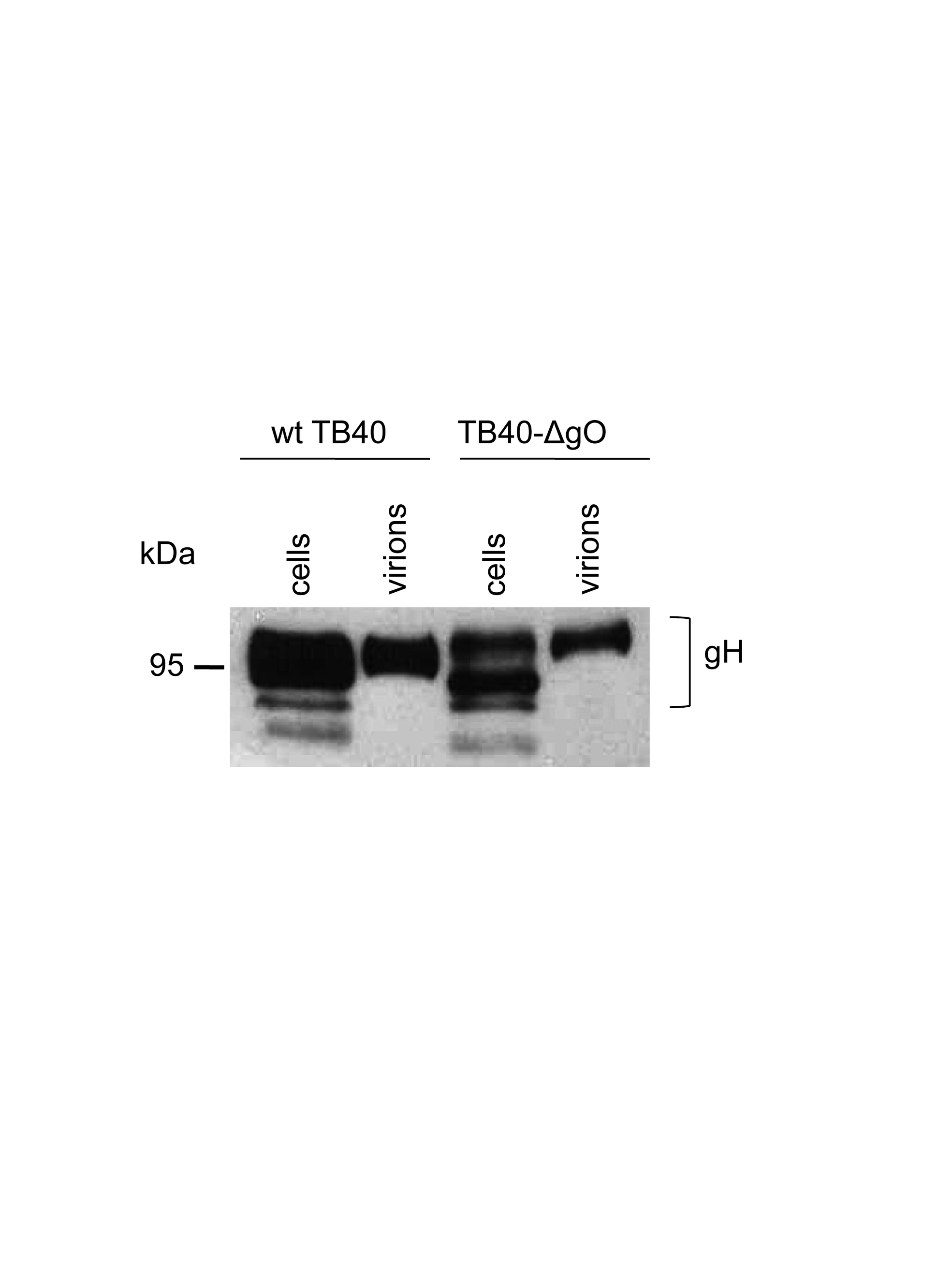

Supplement: S2 Fig — Lysates of HFF infected with wt TB40 or TB40-ΔgO virus and lysates of respective cell-free virions were analyzed by Western blot using an anti-gH antibody. (TIF) [file ppat.1006281.s002.tif]

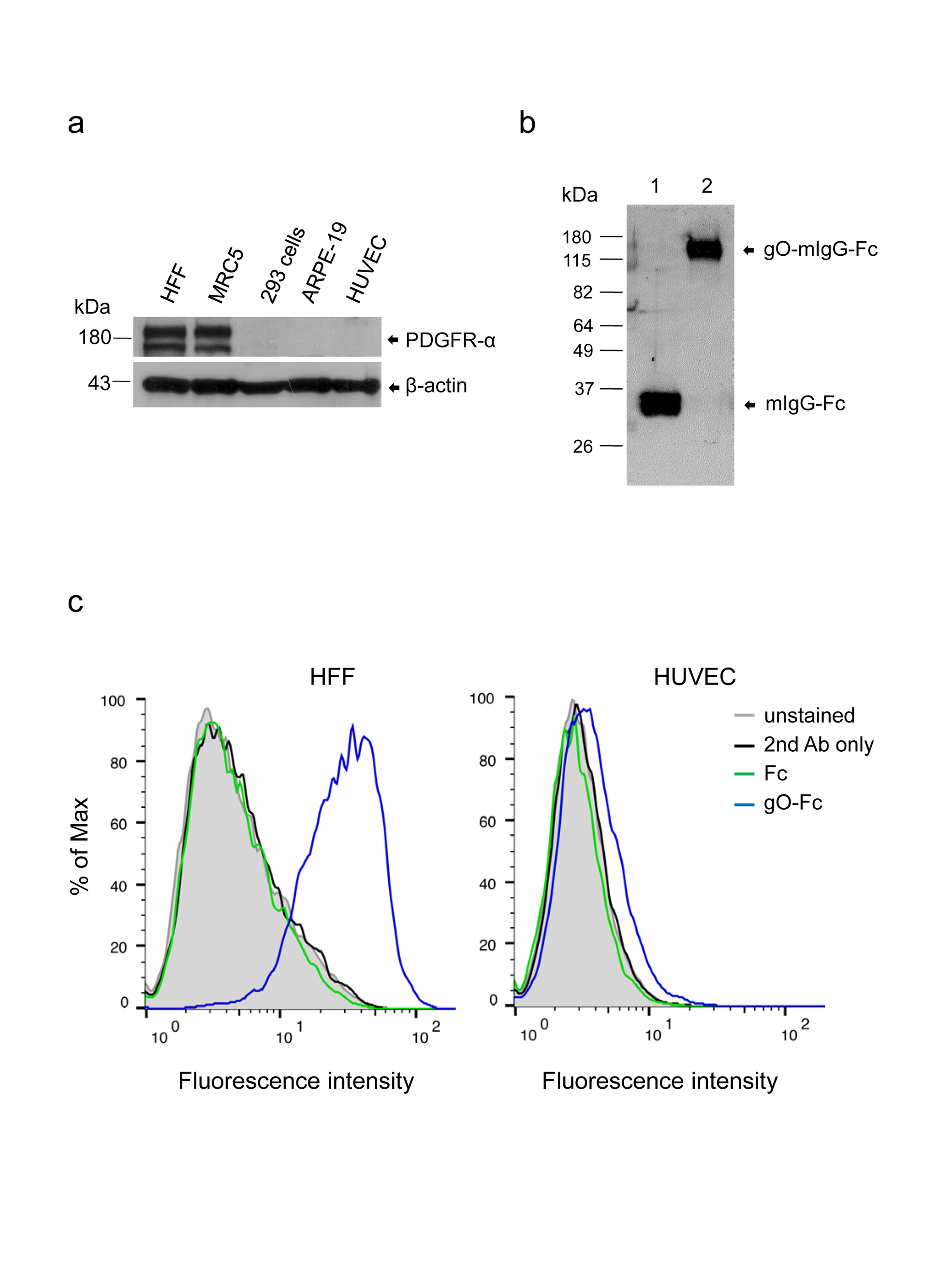

Supplement: S3 Fig — (a) Total cell extracts of HFF, MRC-5, 293 cells, ARPE-19, and HUVEC were analyzed by Western blot for the expression of PDGFR-α and β-actin. (b) 293 cells were transfected with pFUSE-mIgG2B-Fc (1) or pFUSE-gO-mIgG2B-Fc (2). 96 hours after transfection, cell culture supernatants were collected and proteins precipitated with ethanol. Fc fusion proteins were detected by Western blot analysis using a peroxidase-coupled anti-mouse antibody. (c) HFF and HUVEC were co-incubated with equal amounts of purified gO-Fc fusion protein or as a control Fc fusion protein and binding determined by FACS analysis using a Fluor 488-labelled goat anti-mouse IgG antibody. (TIF) [file ppat.1006281.s003.tif]

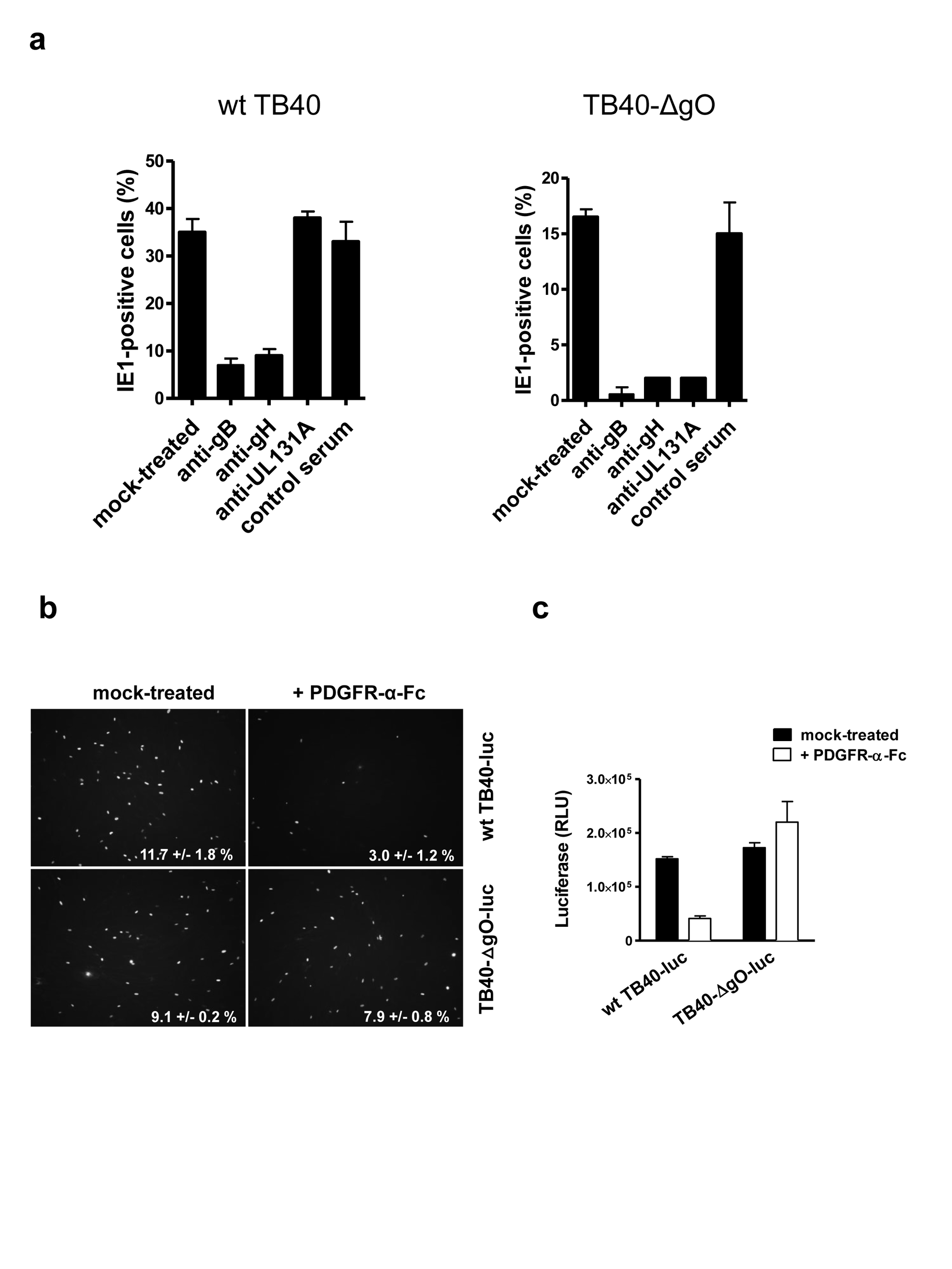

Supplement: S4 Fig — (a) Neutralization of infection with antibodies and (b,c) direct comparison of two methods to quantify HCMV infection of HFF: indirect immunofluorescence staining for HCMV IE1 (b) and luciferase assay (c). (a,b,c) HFF were infected on 96 well plates. Before infection, wt TB40 and TB40-ΔgO viruses were pre-incubated with (a) anti-gB antibodies (SM5-1, 2 μg ml-1), anti-gH antibodies (14-4b), anti-UL131A rabbit antiserum (1:40), control rabbit antiserum (1:40) or medium (mock-treated) or (b,c) PDGFR-α-Fc (300 ng ml-1) or medium (mock-treated) for one hour at 4°C. wt and ΔgO virus inocula were adjusted to result in comparable numbers of infected HFF under mock conditions. Cells were infected using centrifugal enhancement, washed three times with PBS after infection and then incubated for 24 hours before infection was detected by indirect immunofluorescence staining for HCMV IE1 (a,b) or by a luciferase assay (c). Under (b) the percentages of IE1-positive nuclei are indicated. Shown are means +/- SD of representative experiments done in triplicates. (TIF) [file ppat.1006281.s004.tif]

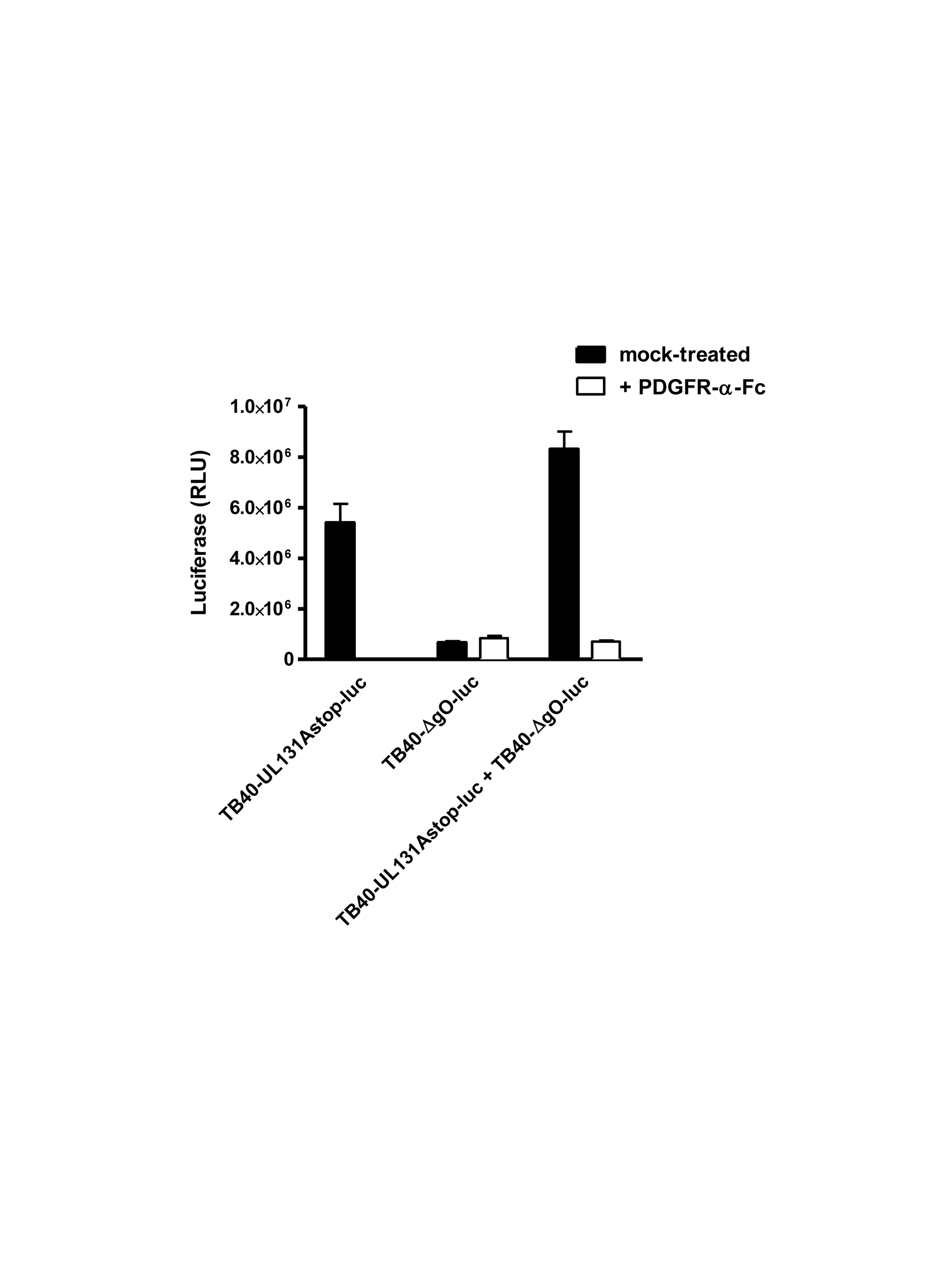

Supplement: S5 Fig — HFF were infected on 96 well plates. Before infection, TB40-UL131Astop-luc or TB40-ΔgO-luc virus or a mixture of both viruses were pre-incubated with 3 μg ml-1 PDGFR-α-Fc or as a control medium for one hour at 4°C. Cells were infected with the virion—PDGFR-Fc mixtures using centrifugal enhancement, washed three times with PBS, and then incubated for 24 hours before infection was determined by a luciferase assay. Shown are means +/- SD of one representative experiment done in triplicates. Inhibition of TB40-UL131Astop-luc by PDGFR-α-Fc was below the detection limit. (TIF) [file ppat.1006281.s005.tif]

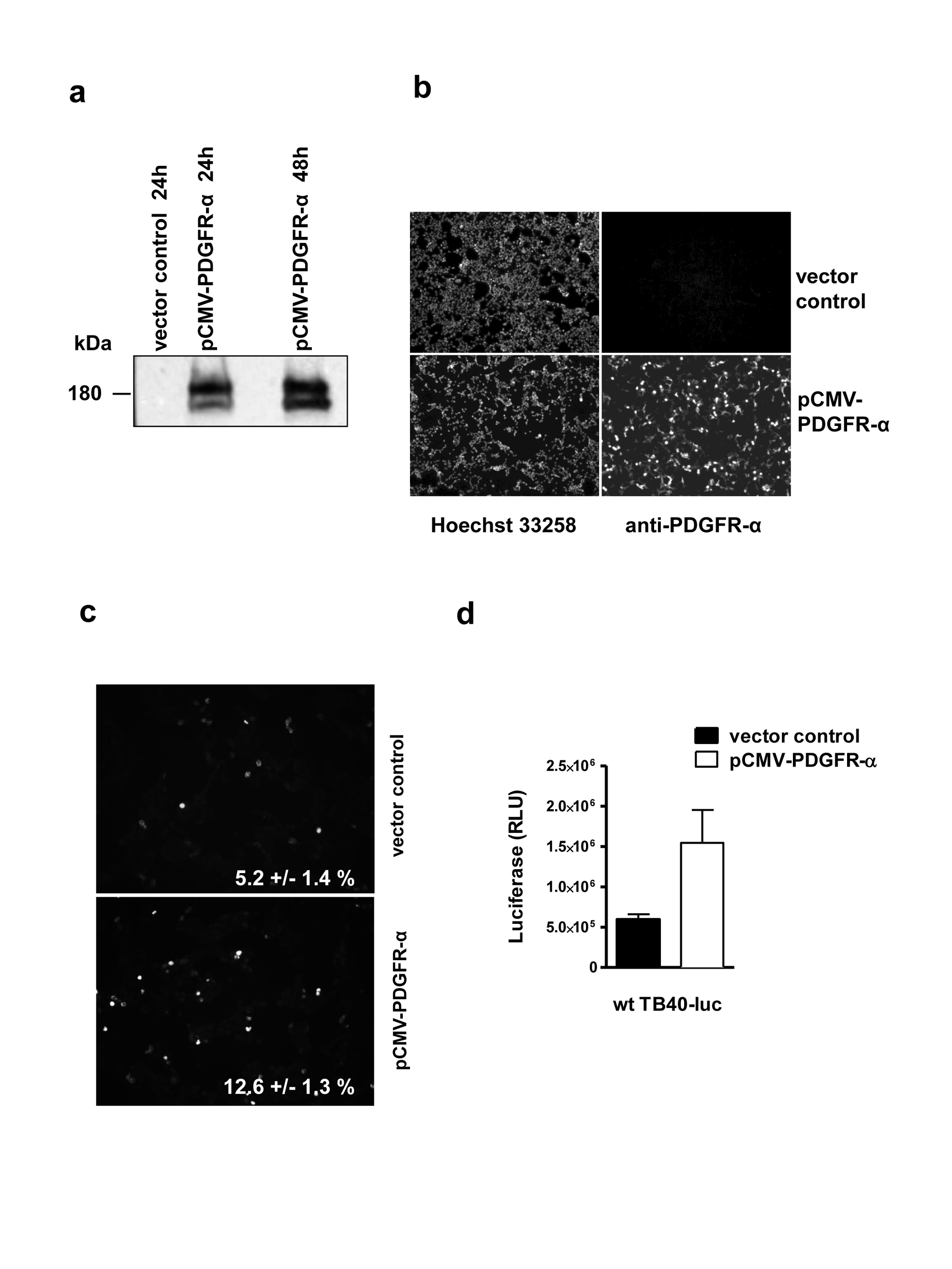

Supplement: S6 Fig — 293 cells were transfected with pCMV-PDGFR-α or a control vector. (a) Total cell extracts were analyzed for PDGFR-α expression by Western blot 24 and 48 hours after transfection. (b) 24 hours after transfection, cells were either stained by Hoechst 33258 to visualize nuclei or by indirect immunofluorescence using a PDGFR-α-specific antibody. (c) and (d) Transfected 293 cells were infected with wt TB40-luc and then incubated for 24 hours before infection was in parallel detected by indirect immunofluorescence staining for HCMV IE1 (c) or a luciferase assay (d). Under (c) the percentages of IE1-positive nuclei are indicated. Shown are means +/- SD of one representative experiment done in triplicates. (TIF) [file ppat.1006281.s006.tif]

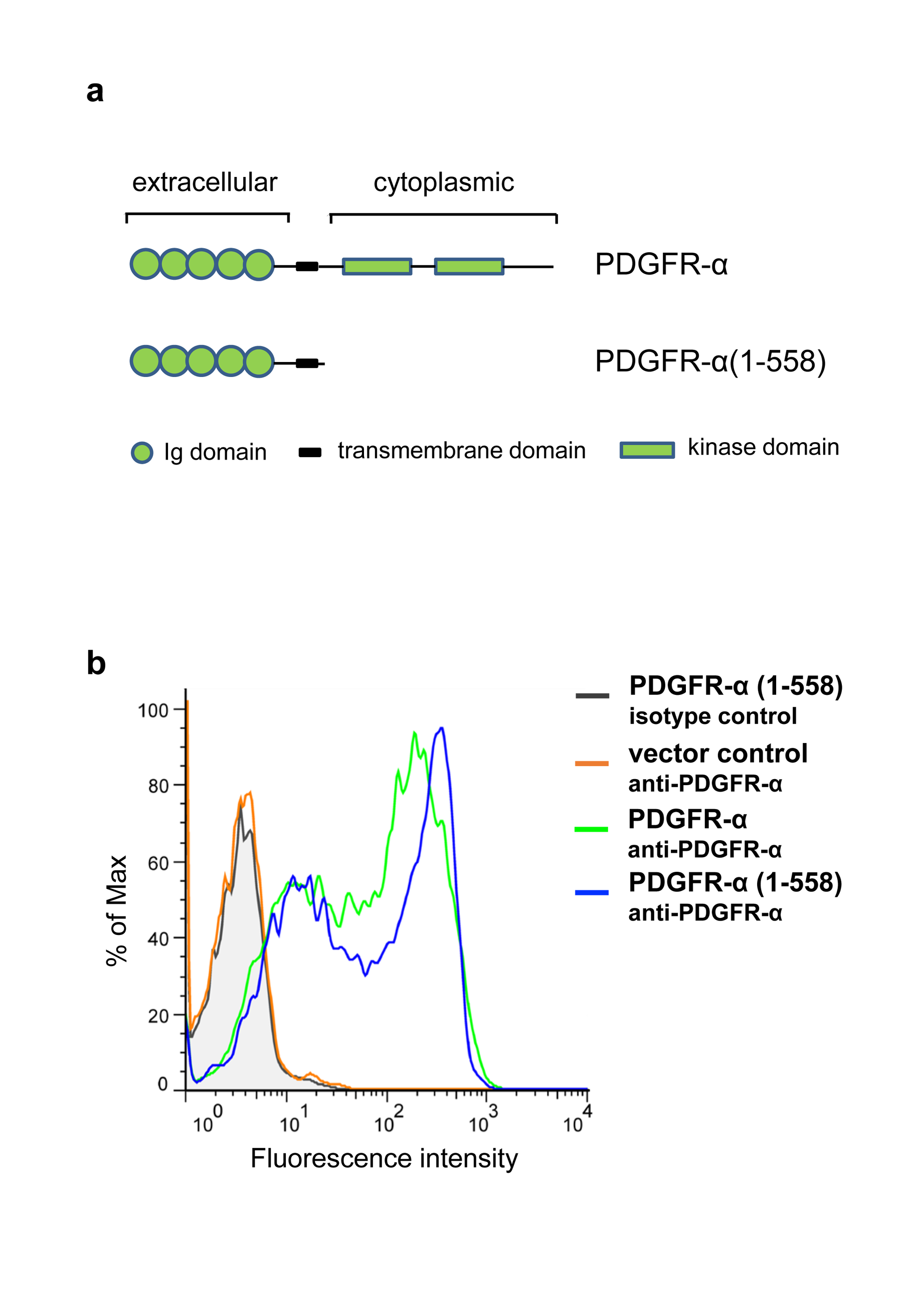

Supplement: S7 Fig — (a) Schematic presentation of full-length and truncated PDGFR-α. The open reading frame of full-length PDGFR-α consists of 1089 amino acids. The last amino acid of truncated PDGFR-α is Arg558. The transmembrane domain ranges from Ala529 to Trp549. (b) 293 cells were transfected with pCMV-PDGFR-α, pCMV-PDGFR-α(1–558), or a vector control. 24 hours after transfection, cell surfaces of transfected cells were stained with an anti-PDGFR-α antibody (35248) or an isotype control and a secondary Fluor 488-labelled anti-mouse antibody and analyzed by FACS. (TIF) [file ppat.1006281.s007.tif]

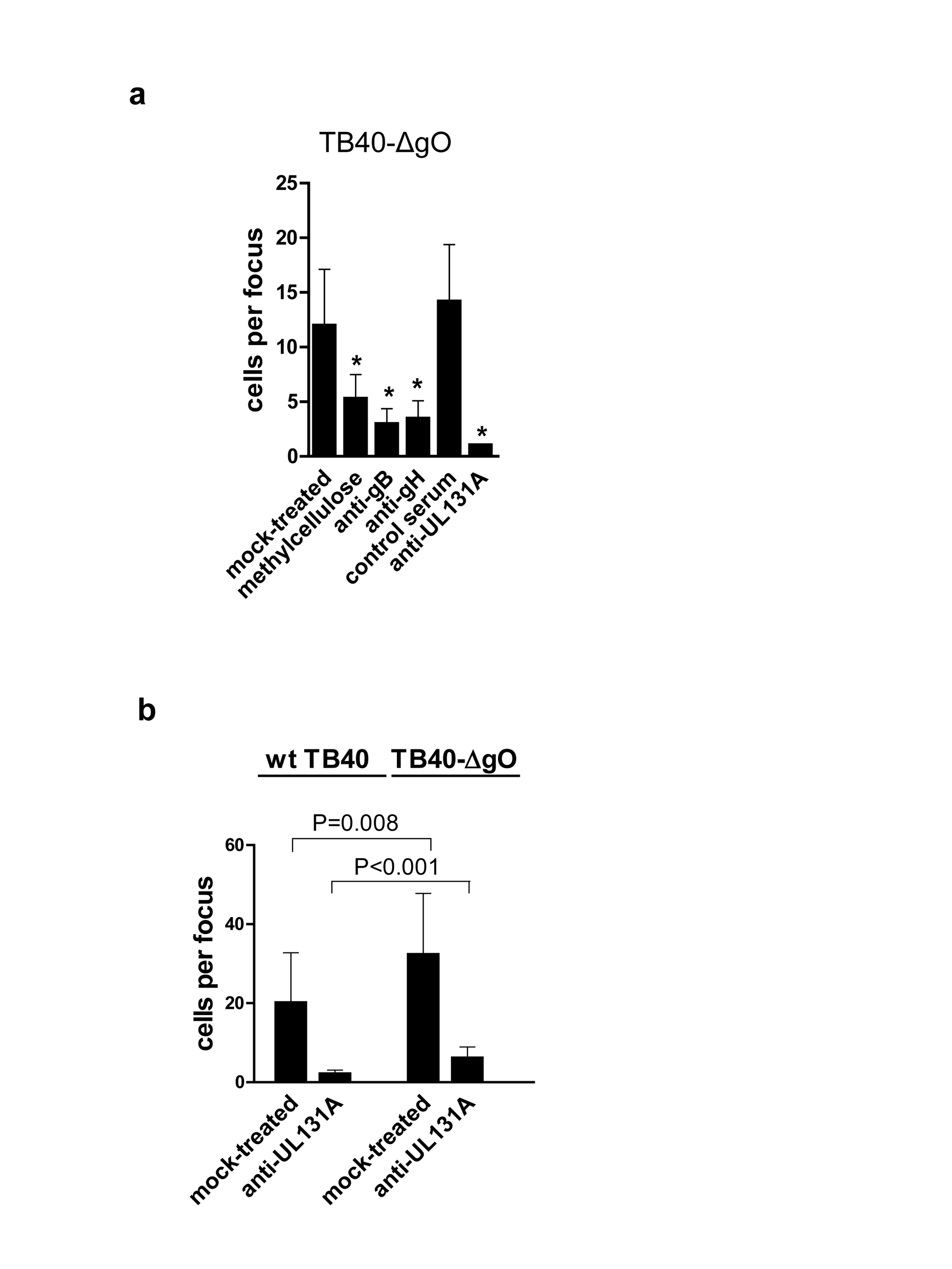

Supplement: S8 Fig — (a) Confluent monolayers of HFF were infected with TB40-ΔgO virus at a very low m.o.i. After infection, cells were either overlaid with methylcellulose or medium containing anti-gB antibodies (SM5-1, 2μg ml-1), anti-gH antibodies (14-4B), anti-UL131A antiserum (1:40), control rabbit antiserum (1:40), or no inhibitor (mock-treated). 5 days later, cells were stained for HCMV IE1 by indirect immunofluorescence and cells per focus counted. For each treatment, at least 12 foci were counted and depicted as means +/- SD. Shown is one representative experiment. Asterisks represent P<0.001 values determined by comparing foci in mock-treated monolayers with foci in monolayers overlaid with methylcellulose or co-incubated with antibodies (Mann-Whitney Rank Sum test). (b) Confluent monolayers of HUVEC were infected with wt TB40 or TB40-ΔgO virus at a very low m.o.i. After infection, cells were either incubated with medium containing anti-UL131A antiserum (1:40) or medium alone (mock-treated). Foci were analyzed as described under (a). P values were determined by comparing foci of wt TB40 and TB40-ΔgO virus infections either under mock conditions or in the presence of anti-UL131A antibodies (Mann-Whitney Rank Sum test). (TIF) [file ppat.1006281.s008.tif]

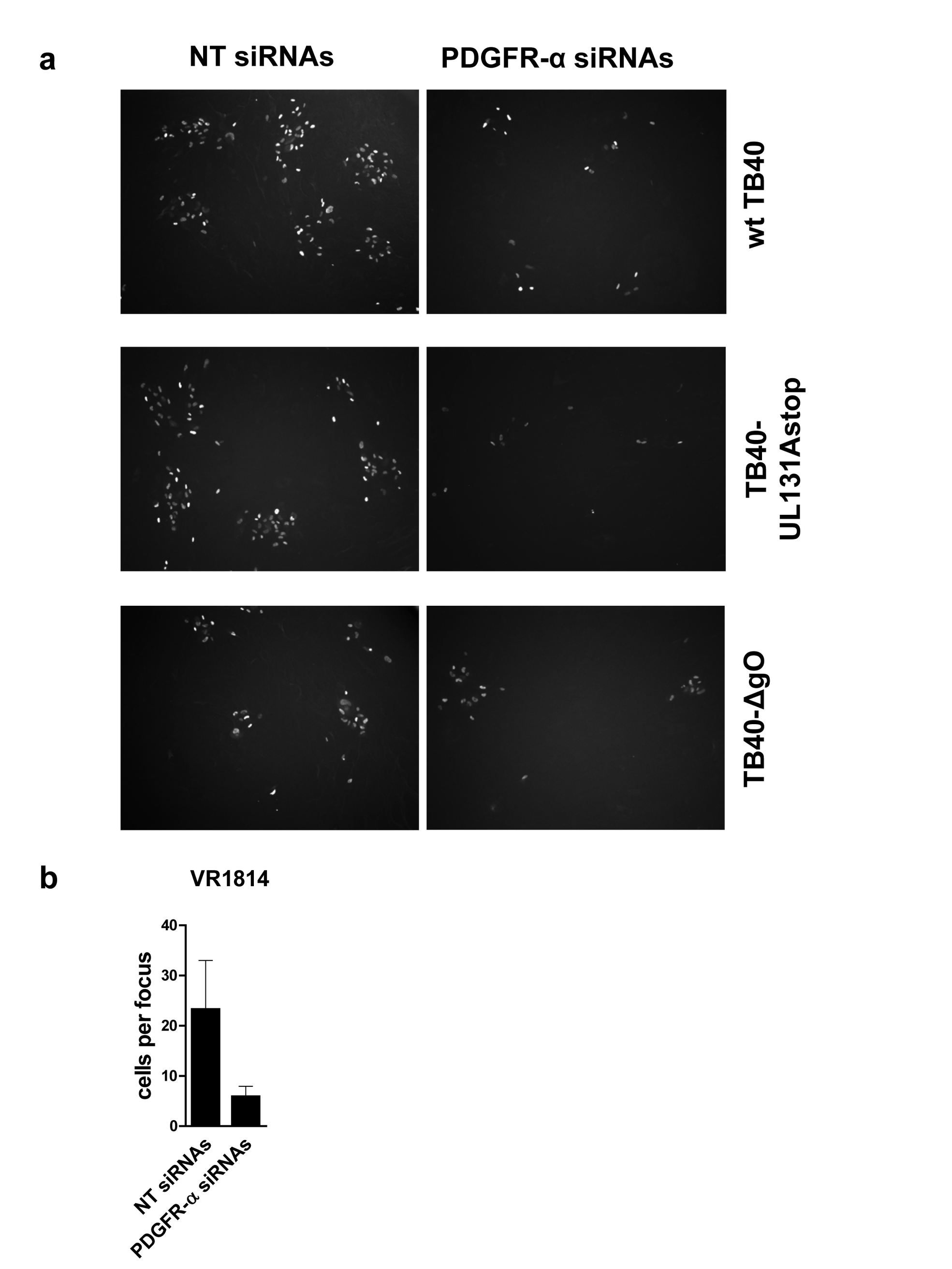

Supplement: S9 Fig — NT siRNA- or PDGFR-α siRNA-transfected HFF 48 hours after transfection were mixed with HFF infected with (a) wt TB40, TB40-UL131Astop or TB40-ΔgO virus or b) VR1814. After adherence, cells were overlaid with methylcellulose. 5 days later, cells were stained for HCMV IE1 by indirect immunofluorescence. (a) Representative stainings of the analysis shown in Fig 6c. (b) One representative experiment for which at least 20 foci were counted and depicted as means +/- SD. (TIF) [file ppat.1006281.s009.tif]
